# ATP is dispensable for *E. coli* DNA replication and eukaryotic helicase activity

**DOI:** 10.1038/s41467-026-73893-5

**Published:** 2026-06-04

**Authors:** Richard R. Spinks, Aleksa Lakic, Celine Kelso, Slobodan Jergic, Olga Yurieva, Zhi-Qiang Xu, Michael E. O’Donnell, Nicholas E. Dixon, Antoine M. van Oijen, Jacob S. Lewis, Lisanne M. Spenkelink

**Affiliations:** 1 Molecular Horizons and School of Science, University of Wollongong, Wollongong, NSW Australia; 2 Laboratory of DNA Replication, Rockefeller University, New York, NY USA; 3 Howard Hughes Medical Institute, Cambridge, MA USA

**Keywords:** Single-molecule biophysics, Enzyme mechanisms, Replisome

## Abstract

Adenosine triphosphate (ATP) hydrolysis is the main cellular source of energy used to drive biochemical reactions that are otherwise energetically unfavourable. The chemical energy stored in phosphoanhydride bonds is released upon hydrolysis of ATP to ADP and is used to drive mechanical work and conformational change. DNA replication is a canonical process in which the multi-enzyme replisome is thought to rely on ATP hydrolysis for its function. Here we show, through single-molecule visualisation of DNA replication by the *Escherichia coli* replisome, that the replicative DnaB helicase does not rely on hydrolysis of ATP in the context of the elongating replisome. Even in the presence of physiologically-relevant concentrations of ATP, dTTP is hydrolysed preferably. Finally, we show that the replicative helicases from *S. cerevisiae*, *D. melanogaster*, and *Homo sapiens* can also use dTTP to unwind DNA. Our observations suggest that replicative helicases across domains of life are ‘flex-fuel’ helicases.

## Abstract (web summary)

Here the authors show that replicative helicases from bacteria to humans can use dTTP instead of ATP for DNA unwinding, and that the E. coli replisome preferentially uses dTTP during DNA replication, challenging textbook models of replication energetics.

## Introduction

**Fig. 1:**
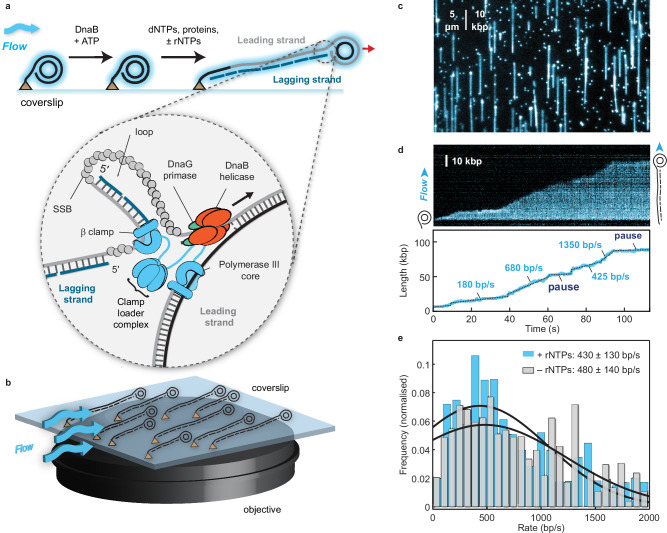
Single-molecule rolling-circle DNA-replication assay. **a** Schematic of the rolling-circle assay. Long products of leading- and lagging-strand DNA are stretched by the laminar flow of buffer. Inset: schematic representation of the *E. coli* replisome. **b** Rolling-circle DNA templates are immobilized on the surface of a microfluidic flow cell mounted on the objective of a TIRF microscope. **c** A typical field of view showing rolling-circle replication products. Scale bar = 10 µm. **d** Kymograph of an individual leading- and lagging-strand replication event. The position of the tip of the DNA corresponds the position of the replisome and the replication rate can be tracked over time using an automated tracking algorithm (blue). Individual rate segments are identified through change-point analysis (black). **e** Distributions of replication rates in the presence (blue, *N* = 58 molecules, 596 segments) and absence (grey, *N* = 58 molecules, 810 segments) of rNTPs, with Gaussian fits. Errors represent s.e.m. Source data are provided on Zenodo^84^.

A model system for DNA replication is the *E. coli* replisome, which requires participation of 12 different proteins to duplicate the chromosome (Fig. 1a, inset)^1,2^. Of these, three components – the DnaB helicase, the DnaG primase and the clamp-loader complex – are known to use ATP in their function.

The replicative helicase DnaB hydrolyses ATP or other ribonucleoside triphosphates, (rNTPs)^3–7^ through its highly-conserved RecA-type ATPase domain^8–11^. The energy from hydrolysis powers DnaB translocation along DNA^4,5,7,12–18^ to unwind the duplex at the replication fork^19,20^. The DnaG primase uses ATP as part of the rNTP pool for the de novo synthesis of RNA primers on the lagging strand^21–26^. The clamp-loader complex uses ATP to load the β_2_ processivity clamp onto DNA^27,28^.

Although these replisomal enzymes have the capacity to use ATP, not all of them completely depend on it. DnaG is a promiscuous primase that is capable of incorporating deoxy-NTPs (dNTPs)^21,23^. Similarly, while the clamp-loader complex predominantly uses ATP, it also accepts dATP as a substitute^29,30^. In contrast, the DnaB helicase is thought to be critically reliant on rNTPs^3–6^.

The assumed dependence of DnaB on rNTPs has led to the widely accepted view that DNA replication progresses in an ATP-dependent manner. However, most ensemble assays do not separate helicase loading from replisome activity and thus do not separately test the ATP dependence of replication elongation (unwinding and synthesis). Here we show, using three different single-molecule replication assays, that efficient replisome activity does not require ATP or other rNTPs. Instead, we show that dTTP is hydrolysed predominantly during DNA replication, even in the presence of ATP. We show that replicative helicases from *E. coli*, *S. cerevisiae*, and *H. sapiens* can use dTTP for unwinding.

## Results

### DNA replication without ATP

Using a flow-cell based single-molecule rolling-circle replication assay (Fig. 1a), we are able to separate helicase loading and replication elongation into discrete steps to test the dependence of replisome-mediated elongation on ATP^31–36^. First, with ATP present, we assembled DnaB from the DnaBC helicase-loader complex onto the rolling-circle DNA template, a 2-kb circular dsDNA with a 5′ flap that mimics the forked DNA found at the site of replication^37^. The helicase-DNA complex was then immobilised on the surface of a microfluidic flow cell. Following helicase loading, and an extensive wash step to remove ATP, replication elongation was initiated by introducing the other replication components necessary for leading- and lagging-strand synthesis.

Leading-strand synthesis displaces ssDNA from the circle, with the liberated ssDNA subsequently acting as a template for lagging-strand synthesis (Fig. 1a). Thus, the rolling-circle design reconstitutes simultaneous leading- and lagging-strand synthesis and results in a single continuously growing dsDNA product. We stretch these growing DNA molecules by laminar flow and visualise a large number of them simultaneously by real-time near-TIRF wide-field imaging, using a dsDNA intercalator as a fluorescent probe (Fig. 1b, c). DNA replication events show distinct periods of constant rates of replication (Fig. 1d, top) Using automated, unbiased tracking and change-point fitting algorithms (Fig. 1d, bottom)^38–40^, we identified and quantified these replication rates. In the presence of ATP (and other rNTPs), we find a mean replication rate of 430 ± 130 bp s^−1^ (mean ± s.e.m.), consistent with previous single-molecule observations (Fig. 1e)^32,33,41^. When we omit the helicase loader DnaC from the DnaB-loading step, the replication efficiency (defined as the number of replication events over the number of DNA templates present at the start) is reduced ~3-fold (Supplementary Fig. 1a). However, omitting ATP from the DnaB-loading step resulted in a complete lack of replication (Supplementary Fig. 1a, b). Similarly, use of an ATPase dead DnaB mutant (DnaB-E262A)^10,42^ in the presence of ATP and DnaC, resulted in a lack of replication, confirming the absolute requirement of ATPase activity of DnaB for replication-competent loading of DnaB (Supplementary Fig. 1a). These findings are consistent with the recent observation that ATP hydrolysis by DnaB is necessary to trigger DnaC release and DnaB ring closure [ref]. Surprisingly, when all rNTPs are omitted from the replication elongation phase, we detect efficient DNA replication with a similar mean rate of 480 ± 140 bp s^−^^1^ (Fig. 1e).

In the absence of rNTPs, we observed sporadic SSB-coated gaps in the lagging-strand product (Supplementary Fig. 2), which we attribute to inefficient Okazaki fragment priming by DnaG^43^ as it incorporates dNTPs less efficiently than rNTPs^21–23^. Notwithstanding these changes in primase behaviour, the key observation is that replisome elongation rates are unaffected by the absence of rNTPs.

### Leading-strand synthesis without ATP

**Fig. 2:**
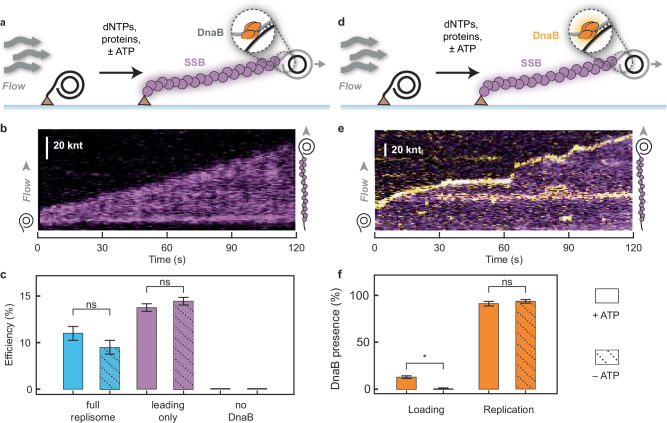
Leading-strand synthesis assay confirms the presence of DnaB irrespective of ATP. **a** The assay is set up as before, with rNTPs and DnaG primase omitted. ssDNA is visualized using fluorescently labelled SSB (purple). **b** Representative kymograph of leading-strand replication. Fluorescent SSB stains the growing ssDNA product (purple). **c** Replication efficiencies for leading- and lagging-strand synthesis (blue), leading-strand synthesis only (purple), and leading-strand synthesis in the absence of DnaB (black), ± ATP. **d** Fluorescent DnaB is added to monitor the presence of DnaB at the site of replication. **e** Representative kymograph showing the presence of DnaB (orange) at the tip of the ssDNA replication product (purple). **f** Quantification of the number of DNA products that have DnaB present at the fork in loading and in replication, ± ATP. Data for DnaB loading is presented in Supplementary Fig. 4. Errors represent s.e.m, from three replicates. Differences were assessed using two-tailed Welch’s t-tests. Source data are provided on Zenodo^84^.

To more closely interrogate the replisomal mechanisms that allow the DnaB helicase to function without ATP, we simplified the reaction to observe only leading-strand synthesis (the components essential for lagging-strand activity were omitted) (Fig. 2a). Since this assay produces ssDNA instead of dsDNA, replication is visualised through imaging of ssDNA-bound fluorescently labelled single-stranded DNA-binding protein, SSB (Fig. 2b)^34,44^. In the presence or absence of ATP, we measure replication rates of 420 ± 60 and 440 ± 60 nt s^−1^, respectively, with similar replication efficiency (Fig. 2c, Supplementary Fig. 3a, b), consistent with previous single-molecule observations that were made in the presence of ATP^31^. This leading-strand assay allows us to ask if the pause frequency and duration depend on the presence of ATP. We found that both were unaffected by the presence of ATP (duration = 7 ± 2 and 7 ± 2 s with and without ATP respectively, Supplementary Fig. 3c; frequency = 2.6 ± 1.2 and 2.2 ± 0.8 pauses per 10 kb with and without ATP respectively).

### The helicase is required for replication

To confirm that the observed replication products are indeed the result of helicase-mediated synthesis, we carried out two important controls. First, the leading-strand synthesis assay was repeated with the simultaneous two-colour visualisation of fluorescent SSB and DnaB helicase labelled in different colours^35^ (Fig. 2d, e). We show fluorescent DnaB is loaded only when ATP is present (Fig. 2f, Supplementary Fig. 4). However, once loaded we detect fluorescent DnaB in all elongating replisomes, irrespective of the presence of ATP during replication (Fig. 2f). For our second control, we repeated the leading-strand synthesis assay without DnaB and found no replication products (Fig. 2c, Supplementary Fig. 5). From these two observations we reaffirm that DnaB is a necessary part of the replisome but does not require ATP to support replication elongation.

### SSB does not contribute to unwinding

**Fig. 3:**
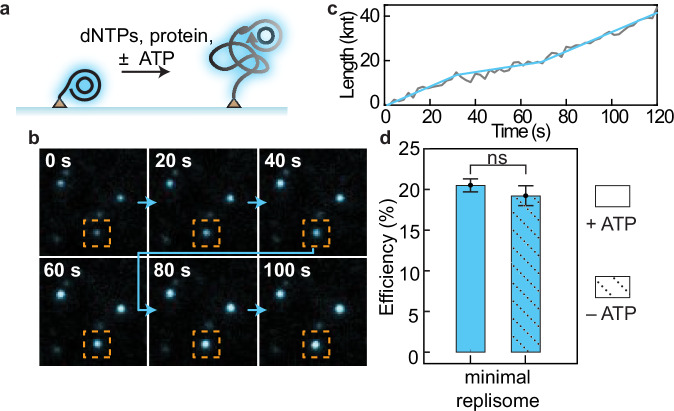
Minimal-replisome assay provides evidence that SSB binding does not contribute to unwinding. **a** Schematic of the assay. In the absence of flow, the newly synthesized DNA forms a compact coil of ssDNA. **b** Montage showing the increase in size and intensity of three individual DNA molecules undergoing replication. **c** Intensity is converted into length and plotted as a function of time (grey) for the boxed molecule in b, and rate segments determined by change-point fitting (blue). **d** Efficiency of replication by the minimal replisome with and without ATP. Errors represent s.e.m, from three replicates. Differences were assessed using two-tailed Welch’s t-tests. Source data are provided on Zenodo^84^.

Next, we sought to determine which energy source, if not helicase-mediated ATP hydrolysis, is the predominant driver of the replisome. SSB is known to have passive unwinding activity whereby it binds to ssDNA that is transiently exposed at a ssDNA-dsDNA fork due to thermal breathing of the dsDNA^45,46^. The bound SSB then potentially acts as a ratchet, preventing the reannealing of the ssDNA. Although SSB is not necessarily required for leading-strand synthesis in the presence of ATP^31^, it is conceivable that SSB binding rather than DnaB helicase activity enables fork progression under conditions without ATP. To exclude this possibility, we designed a minimal replication assay in which we also omit SSB from leading-strand rolling-circle replication. We carried out the assay in the absence of flow (Fig. 3a) so that the exposed ssDNA synthesis product forms an entropic coil with high local concentration of ssDNA, which can be stained and visualied using the low-affinity binding of the DNA fluorescent stain to ssDNA. We monitor replication through an increase in intensity of the stained DNA product (Fig. 3b, d). The assay was calibrated using the intensity of a ssDNA template of known length (Supplementary Fig. 6a, b). Using change-point analysis we find a rate of replication of 340 ± 70 nt s^−1^ in the presence of ATP (Supplementary Fig. 6c). This rate is comparable to previously reported rates from single-molecule experiments in the absence of SSB^31^. Again, we see that the replication efficiency (Fig. 3d) and replication rates (Supplementary Fig. 6c) are unaffected by the absence of ATP. These results indicate that SSB is not responsible for strand separation.

### DnaB can hydrolyse dTTP during replication

**Fig. 4:**
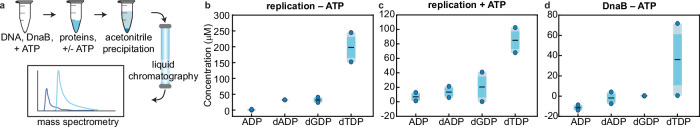
ATPase assay shows minimal nucleotide hydrolysis by DnaB during replication. **a** Schematic of the LC-MS assay. DnaB is loaded in the presence of ATP. The reaction is initiated in the presence or absence of ATP. After 30 min the reaction is quenched with acetonitrile and the presence of nucleotides is analysed via LC-MS (see Methods). **b** Amount of ADP, dADP, and dTDP generated after 30 min of replication in the absence of 1.25 mM ATP (see methods for concentrations of the other nucleotides). dTTP is preferentially hydrolysed. **c** Amount of ADP, dADP, and dTDP generated after 30 min of replication in the presence of 1.25 mM ATP. dTTP is still preferentially hydrolysed. **d** Amount of ADP, dADP, and dTDP generated after 30 min of unwinding by DnaB in the absence of ATP. Source data are provided on Zenodo^84^.

Finally, we consider the possibility that DnaB might hydrolyse dNTPs when associated with the replisome. Therefore, we measured hydrolysis of ATP and dNTPs by measuring the presence of ADP and dNDPs in an LC-MS experiment at the end of a replication reaction (Fig. 4a). We load DnaB in the presence of ATP in a very small volume, before initiating the replication reaction (in the absence of rNTPs) in a 20-fold larger volume (Fig. 4a). We quench the reaction at 30 min and precipitate out all the proteins and DNA, allowing us to measure the presence of nucleotides in the supernatant. First, as a control, we measured the appearance of ADP in a reaction with DnaB, ATP, in the presence of single-stranded DNA. We see ATPase activity similar to literature values (Supplementary Fig. 7)^11^. Next, we measured the appearance of ADP and dNDPs in the replication reaction. As expected, since ATP is present at very low concentrations, we do not see generation of ADP (Fig. 4b). Unexpectedly, we see generation of dNDPs, in particular dTDP (Fig. 4b). These results suggest that, during DNA replication, DnaB is a flex-fuel helicase, able to hydrolyse different d/rNTPs. These findings are consistent with recent findings showing rapid and processive translocation by DnaB in the presence of different d/rNTPs^47^.

### Even with ATP added it is not hydrolysed

A situation where an active replisome is starved of all ATP would not be a common occurrence in the cell. Although our experiments demonstrate replication without ATP, they provide no measure of the frequency at which DnaB might hydrolyse ATP when the nucleotide is available during replication. Therefore, we measured the ATPase activity of DnaB in the presence of physiologically relevant concentrations of ATP. Strikingly, the ATPase activity is still minimal (Fig. 4c). In fact, ATPase activity is 15 fold lower than in our control (Supplementary Fig. 7d) and ~20 fold lower than the value calculated based on basic properties of the replication process (assuming DnaB unwinds 2 nt for every ATP hydrolysed^11^ and a replication rate of 500 bp∙s^−1^ (Fig. 1e), and a replication efficiency of 60%).

We supported these findings by repeating the measurements using a fluorescence polarisation assay (Supplementary Fig. 8). Again, we see that ATPase activity during replication is much lower than expected (Supplementary Fig. 8c). In fact, this reaction did not hydrolyse significantly more ATP than a Pol III strand-displacement reaction in which DnaB is omitted (Supplementary Fig. 8c, e). We confirmed that efficient DNA replication occurred in all replication experiments, by running the DNA products from these assays on an agarose gel (Supplementary Fig. 8d, e). Interestingly, we again see that dTTP is hydrolysed preferentially (Fig. 4c).

### Hydrolysis of dTTP is conserved for replicative helicases

**Fig. 5:**
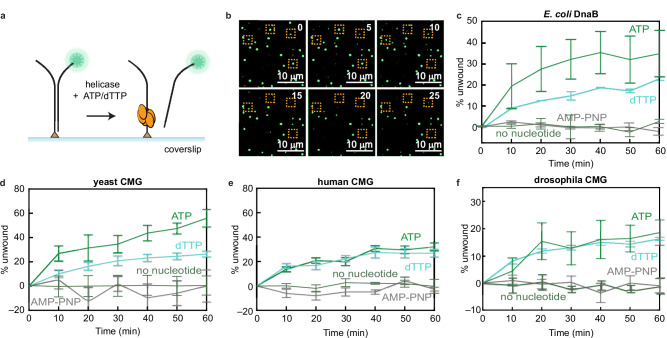
Replicative helicases can use dTTP for unwinding. **a** Schematic representation of the DNA unwinding assay. A forked, fluorescently labelled DNA template is immobilised on the surface of a microscope coverslip. Unwinding of the substrate by the helicase will result in a loss of the fluorescently labelled strand. **b** Montage showing the disappearance of spots upon unwinding by DnaB. **c** The percentage of templates unwound by DnaB as a function of time in the presence of ATP (green) and dTTP (cyan). No-nucleotide (dark green) and AMP-PNP (grey) controls do not show unwinding. **d** The percentage of templates unwound by yeast CMG as a function of time in the presence of ATP (green) and dTTP (cyan). No-nucleotide (dark green) and AMP-PNP (grey) controls do not show unwinding. **e** The percentage of templates unwound by human CMG as a function of time in the presence of ATP (green) and dTTP (cyan). No-nucleotide (dark green) and AMP-PNP (grey) controls do not show unwinding. **f** The percentage of templates unwound by drosophila CMG as a function of time in the presence of ATP (green) and dTTP (cyan). No-nucleotide (dark green) and AMP-PNP (grey) controls do not show unwinding. Error bars represent s.e.m. Source data are provided on Zenodo^84^.

Our observation that DnaB can hydrolyse dTTP during DNA replication, led us to investigate if it could hydrolyse dTTP during DNA-unwinding in isolation. We set up a simple single-molecule assay where we load short forked DNA substrates on the surface of our microfluidic flow cell. (Fig. 5a). We then introduced DnaB (complexed with its loader DnaC) in the presence of ATP. Next, we initiated the reaction by loading either ATP or dTTP. If unwinding occurs, fluorescently-labelled strand will be displaced, resulting in a loss of fluorescence. We see that DnaB can unwind the template in the presence of dTTP as well as ATP (Fig. 5c, Supplementary Table 2).

Other helicases are known to be flex-fuel helicases. The bacteriophage T7 helicase, gp4, is known to hydrolyse dTTP preferably^48,49^. The mitochondrial replicative helicase, TWINKLE, is also known to hydrolyse a variety of nucleotides, including dTTP^50^. Therefore, we wondered if this behaviour is conserved more broadly for replicative helicases. We repeated our single-molecule unwinding assay using CMG from yeast (*S. cerevisiae*)^51^, humans (*H. sapiens*)^52^, and flies (*D. melanogaster*)^47^. Interestingly, we see that all three are able to unwind DNA using dTTP (Fig. 5d–f). While the unwinding activity of yeast CMG is slightly reduced with dTTP compared to ATP (Fig. 5e), the human and fly CMGs show similar unwinding activity using either nucleotide (Fig. 5f)^4,10,11^. Consistent with our findings, *D. melanogaster* CMG has recently been shown to translocate processively in the presence of dNTPs^47^.

## Discussion

Taken together, our data suggest that hydrolysis of ATP by the replicative helicase is not required for DNA strand separation. Instead, we demonstrate that dTTP hydrolysis predominantly drives DNA replication in *E. coli*. This result challenges the longstanding assumption that ATP hydrolysis is the sole cofactor powering the unwinding of DNA during replication^1,11,53–56^. In our assays (Fig. 4b, middle) we have a 3-fold excess of ATP, yet dTTP is preferentially used by ~16-fold. In live cells ATP is in a ~ 10-fold excess over dTTP^57^. Assuming a simple linear relation between ATP/dTTP ratio and nucleotide usage, this still implies ~5-fold greater use of dTTP. However, the exact contribution of ATP and dTTP hydrolysis to replisome progression in vivo remains to be established.

Unwinding (Fig. 5c) and translocation assays^47^ do not show the strong dTTP preference observed during replication, indicating that this is a replisome-engaged behaviour. The mechanism underlying this preference is currently unknown and will require future investigation. Notably, ATP is still required during the helicase loading step. While the mechanistic basis for this difference in nucleotide requirement remains unclear, ATP binding or hydrolysis may promote a conformational transition that places DnaB in an elongation-competent state.

We further show that DnaB also uses dTTP when unwinding DNA outside the context of the replisome. Interestingly, the rate of unwinding (~2 nt/s^9,11,58^) and the rate of strand-displacement synthesis (~100–150 nt/s, Supplementary Fig. 8e^58^) are both much smaller than the rate of DnaB-mediated replication (Fig. 1e, Supplementary Fig. 3b). This indicates a strong cooperativity between the helicase and the polymerase. It has been shown previously using the simpler bacteriophage T7 replisome that the replicative polymerase and helicase each stimulates the activity of the other, indicating an active role of the polymerase in driving DNA replication at maximal rates^59^. Further, it was proposed that the T7 helicase and polymerase cooperatively participate in DNA unwinding, through partial melting of the DNA by each enzyme^60^. Moreover, a cryo-EM structure of the T7 replisome shows the polymerase positioned perpendicular to the helicase at the DNA fork junction, also implying a cooperative approach to DNA unwinding^61^. While structures of sub-complexes of the *E. coli* replisome exist^28,62–64^, there is currently no structure of the full *E. coli* replication complex. However, it seems likely that DnaB provides the platform that dictates the architecture of the *E. coli* replisome to facilitate strand separation. Previous observations of a dependence of DnaB and polymerase activity on the presence of each^31,61^ and the modulation of DnaB helicase unwinding activity by DNA geometry are consistent with this model^19^.

We extend our finding of a flex-fuel helicase to yeast and human replicative helicases. Replicative helicases in many bacteriophages have been known to hydrolyse multiple rNTPs and dNTPs^48,49,65–68^. However, to our knowledge, this behaviour has not been reported for bacterial and eukaryotic replicative helicases. The conservation of this behaviour across evolutionary domains suggests that the flexibility to use d/rNTPs may represent a fundamental property of replicative helicases, independent of species or cellular context. The ability for the helicase to hydrolyse different nucleotides may provide cells with the flexibility to operate under different cellular conditions and continue functioning regardless of changes in nucleotide balance. Such flexibility of the replisome to function under different conditions has been emerging through other single-molecule studies in the last decade^2,69–71^.

The observations presented here suggest future studies to investigate the function of other helicases whose activity may be influenced by the translocation of other proteins on DNA and those involved in DNA and RNA metabolism.

## Methods

### Proteins

DNA replication proteins were produced as described previously: the β_2_ sliding clamp^72^, SSB^73^, AF647- and AF488-labelled SSB-K43C^34^, the DnaB_6_(DnaC)_6_ helicase–loader complex, DnaC loader and AF647-labelled DnaB_6_H201C^35^, DnaG primase^74^, the τ_3_δδ’χψ clamp loader^31^, and Pol III αεθ core^33,75^, S. cerevisiae CMG^76^.

### Single-molecule rolling-circle experimental design

Construction of the 2030 bp template used for most rolling-circle assays has been described^37^. Microfluidic flow cells were prepared as described in ref. ^77^. Briefly, a PDMS flow chamber was placed on top of a PEG-biotin-functionalised microscope coverslip. To help prevent non-specific interactions of proteins and DNA with the surface, the chamber was blocked with buffer containing 50 mM Tris-HCl pH 7.5, 50 mM KCl, and 2% Tween-20. The chamber was placed on an inverted microscope (Nikon Eclipse Ti-E) with a CFI Apo TIRF 100x oil-immersion TIRF objective (NA 1.49, Nikon) and connected to a syringe pump (Adelab Scientific) for flow of buffer. Reactions were carried out at 31 °C, maintained by an electrically heated chamber (Okolab). Double-stranded DNA was visualised in real time by staining it with 150 nM SYTOX Orange (Invitrogen) excited by a 514 nm laser (Coherent, Sapphire 514-150 CW) at 150 μW cm^−^^2^. The red-labelled SSB was excited at 700 μW cm^−2^ with a 647 nm laser (Coherent, Obis 647-100 CW). Imaging was done with an EMCCD camera (Hamamatsu) through Nikon Elements 4.30 software. Signals were separated via dichroic mirrors and appropriate filter sets (Chroma).

### Leading- and lagging-strand replication assay

Conditions for simultaneous leading- and lagging-strand DNA replication were adapted from previously described methods^33–35^. Briefly, 4 nM DnaB_6_(DnaC)_6_ was pre-loaded onto 20 pM rolling-circle DNA template by incubation at 37 °C for 30 s in replication buffer (25 mM Tris-HCl pH 7.9, 50 mM potassium glutamate, 10 mM Mg(OAc)_2_, 40 μg ml^−1^ bovine serum albumin, 0.1 mM EDTA and 5 mM dithiothreitol) + 1 mM ATP. This mixture was loaded into the flow cell at 70 μl min^−1^ for 60 s and then at 10 μl min^−^^1^ for 8 min. To remove any unbound DNA and ATP from solution, the flow cell was vigorously washed with 100 flow-cell volumes of replication buffer. The reaction buffer consisted of 60 μM of each dNTP, and 1.25 mM ATP 250 μM each of CTP, GTP and UTP (when indicated) in replication buffer. Pol III* was assembled in situ by incubating τ_3_δδ’χψ clamp loader (410 nM) and Pol III αεθ core (1.2 μM) in reaction buffer at 37 °C for 90 s. Replication was initiated by flowing in the reaction buffer containing 3 nM Pol III*, 30 nM β_2_, 75 nM DnaG, and 20 nM SSB_4_ at 10 μl min^−1^.

### Leading-strand replication assay

Unlabelled and labelled DnaB_6_(DnaC)_6_ were pre-loaded on the rolling-circle DNA template before immobilisation of the helicase–DNA complex on the surface of the microfluidic flow cell, as described above. To remove any unbound template and ATP from solution, the flow cell was washed with 100 flow-cell volumes of replication buffer. The reaction buffer contained 60 μM of each dNTP and 1 mM ATP as indicated. Pol III* was assembled in situ by incubating τ_3_δδ’χψ clamp loader (410 nM) and Pol III αεθ core (1.2 μM) in replication buffer at 37 °C for 90 s. Replication was initiated by flowing in the reaction buffer containing 3 nM Pol III*, 30 nM β_2_, and 20 nM labelled SSB_4_ at 10 μl min^−1^.

### Quantification of the length of SSB-coated ssDNA

We estimated the length of SSB-coated ssDNA in our assay based on the intensity of the labelled SSB. We divided the total intensity of 16 leading-strand synthesis products by the intensity of a single SSB. We assumed the SSB binds in the 35-mode to obtain a conversion factor of 800 ± 400 nt pix^−1^ (Supplementary Fig. 4a).

### DnaB loading quantification

Labelled DnaB_6_(DnaC)_6_ at 4 nM was pre-loaded onto 20 pM rolling-circle DNA template as described above. This complex with 150 nM SYTOX orange was loaded into the flow cell at 70 μl min^−1^ for 60 s and then at 10 μl min^−1^ for 8 min. We imaged SYTOX stained DNA and labelled DnaB sequentially. We determined the position of DNA and DnaB foci using a peak-finding algorithm that fits a 2D Gaussian to give sub-pixel localisation. Foci were defined as colocalised when they were within 2 pix of each other. Colocalisation by chance was calculated based on area overlap^33^.

### Minimal replication assay

DnaB_6_(DnaC)_6_ was pre-loaded on the rolling-circle template before immobilisation of the helicase-DNA complex on the surface of the microfluidic flow cell, as described above. To remove any unbound template and ATP from solution, the flow cell was washed with 100 flow-cell volumes of replication buffer. The reaction buffer contained 60 μM of each dNTP and 1 mM ATP as indicated. Pol III* was assembled in situ by incubating τ_3_δδ’χψ (410 nM) and Pol III cores (1.2 μM) in replication buffer at 37 °C for 90 s. Replication was initiated by flowing in the reaction buffer containing 3 nM Pol III*, 30 nM β_2_, and 150 nM SYTOX orange at 10 μl min^−1^.

### Calibration of SYTOX intensity as a function of ssDNA length

To quantify the rates of replication in the minimal assay, the intensity of SYTOX stained ssDNA was calibrated using M13 ssDNA (Supplementary Fig. 6). A 66-mer 5’-biotin-(T)_36_ AAT TCG TAA TCA TGG TCA TAG CTG TTT CCT-3’ (Integrated DNA Technologies) was annealed to M13mp18 ssDNA (Guild Biosciences). This ssDNA template was loaded on the flow cell in reaction buffer and imaged using conditions identical to those used during the minimal replication assay. Using the average intensity measured in this assay and the known length of M13mp18 (7429 nt), an intensity per nt conversion factor was obtained.

### Quantification and statistical analysis of single-molecule experiments

All analysis was done with ImageJ 1.51w and MATLAB 2016b using in-house built plugins. The position of the tip of growing rolling-circle products was tracked as a function of time using an automated tracking algorithm. Individual rate segments were identified using an unbiased change-point algorithm^38–40^. Pauses were defined as any rate segments < 50 nt s^−1^. In the minimal replication assay, single-molecule trajectories were obtained by tracking the intensity of the SYTOX stained ssDNA product over time. The change of intensity in time (rate) was converted to nt s^−1^ using the M13 ssDNA calibration. The rate histograms were weighted by the length of the segments to reflect the higher confidence for longer segments^78^. All single-molecule experiments were carried out in triplicate. The number of molecules or events analysed is indicated in the text or figure legends. Errors reported in this study represent the standard error of the mean (s.e.m.) or the error of the fit, as indicated in the text or figure legends.

### LC-MS assays

Nucleotide diphosphates and triphosphates (ATP, dATP, ADP, dADP, dGDP, dGTP, dTTP, dTDP) were purchased from Sigma. Acetonitrile (LCMS grade) and Formic acid (LC_MS grade) were purchased from Thermo Fisher Scientific. Ammonium Formate (LC-MS grade) from Sapphire Bioscience.

Chromatographic separations were carried out with a Water™ Acquity Ultra Performance Liquid Chromatography system (UPLC) using a Waters Atlantis™ Premier BEH Z-HILIC (2.1 × 50 mm, 1.7 µm) column fitted with a VanGuard™ Pre-Column (2.1 × 5 mm, 1.7 μm). The separation of nucleotides was achieved with a binary solvent gradient of 300 mM ammonium formate pH 4.0 (adjusted with formic acid, mobile phase A) and acetonitrile (mobile phase B) at 0.5 ml/min. Sample were maintained at 10 °C prior to injection. The injection volume was 10 µL and column temperature was set to 50 °C. The elution gradient was as follows: initial concentration 75%B, 75–60%B from 0 to 6 min, 60 to 40%B from 6 to 7 min, 40%B from 7 to 9 min, 60 to 75%B from 9.1 min and a reconditioning step at 75%B from 9.1 to 20 min. All the nucleotides eluted were eluted within 6 min.

Detection and quantification was carried out by mass spectrometry using a QTRAP5500 hybrid triple quadrupole linear ion trap mass spectrometer (Sciex, USA) with a turbo ion spray source. The detection was done in positive-ion mode using the multiple-reaction-monitoring (MRM) mode. The source parameters were set as follows: curtain gas (CUR) 20 psi, spray voltage (IS) 5500 V, temperature (TEM) 600 ^o^C and auxiliary gases 1 and 2 both set to 20 psi (GS1 and GS2). Fragmentation optimisation for each compound was done experimentally by direct infusion of individually prepared standard solution (50 µg/ml in water) at a flow rate of 5 µl/min into the 0.5 ml/min LC flow (65%B composition). Mass spectrometry parameters were optimised for each individual compound to obtain ionisation and fragmentation voltages for maximum signal. Supplementary Table 1 provides the optimised parameters for the declustering potential (DP), collision cell entrance and exit potential (EP and CXP, respectively) and collision energy (CE) for each compound.

Quantification was carried out using a 7-point calibration curve using working standards of the mixed analytes ATP, ADP and dADP at 2, 10, 20, 30, 40, 50, and 100 mM in 25% water/75% acetonitrile (other compounds were detected but not quantified). Both the parent ion (Q1 *m/z*, Supplementary Table 1) and most intense fragments (Q3 *m/z*, Supplementary Table 1) were used for detection and quantification. Analyst 1.6.2 (ABSciex, Framingham MA, USA) was used for both data acquisition and data analysis.

Replication and unwinding reactions were set up in replication buffer and contained 2 nM rolling-circle template. The unwinding reaction contained 1.25 mM ATP and was initiated by addition of 10 nM DnaB_6_(DnaC)_6_. The replication reaction in the presence of ATP contained 1.25 mM ATP, 400 μM of each dNTP, 10 nM DnaB_6_(DnaC)_6_, 30 nM αεθ, 10 nM τ_3_δδ’χψ, 30 nM β_2_, 75 nM DnaG, 1 µM SSB_4_. To estimate the ability of DnaB to hydrolyse dTTP we loaded DnaB in the presence of ATP (50 µM ATP, 10 nM DnaB) in a small volume. After incubation at 37 °C for 5 min, the rest of the replisomal components and dNTPs were added to initiate the reaction (400 μM of each dNTP, 30 nM αεθ, 10 nM τ_3_δδ’χψ, 30 nM β_2_, 75 nM DnaG, 1 µM SSB_4_) in a final volume of 100 µl.

At t = 0 min (immediately after initiation) 8 µl of the solution mixture was taken out and added to a vial containing 72 μl of water and 240 μl of acetonitrile to result in diluted reaction mixture in 25% aqueous and 75% acetonitrile. Enzymes and DNA precipitated out immediately quenching the reaction. 10 μl of the supernatant was injected via LCMS for analysis. At t = 30 min the reactions were sampled and analysed again as described above. Supplementary Fig. 7a shows example spectra.

Standard solutions for ADP, ATP and dADP were prepared to quantify these nucleotides in the reaction mixture. Individual solutions of ADP, ATP and dADP (100 mM in milliQ) were prepared and stored at −80 ^o^C until required. From these, a 1 mM fresh mix standard containing each compound was prepared (1 ml) by diluting appropriate amount of the stock solution with water. Working standards were then prepared from the mix standard solution by dilution with water/acetonitrile to obtain the following concentrations: 2, 10, 20, 30, 40, 50 and 100 μM in 25% water/75% acetonitrile. Working standards were analysed under the same conditions as described above. Calibration curves were obtained by plotting the peak area *vs*. concentration for each analyte (Supplementary Fig. 7b). Concentrations of ATP, ADP and dADP in the reaction mixtures at each time point tested were determined against their respective calibration curves. The amount of nucleotide generated was determined as the difference between the amount of nucleotide present at t = 0 min *vs*. t = 30 min. When ATP was not present, no generation of ADP was detected (Supplementary Fig. 7c).

### Fluorescence polarisation assays

Generation of ADP was measured using a fluorescence polarisation assay (Supplementary Fig. 8a; Transcreener ADP^2^ FP Assay, Sigma)^79^. All reactions were carried out in replication buffer (25 mM Tris-HCl pH 7.9, 50 mM potassium glutamate, 10 mM Mg(OAc)_2_, 40 μg ml^−1^ BSA, 0.1 mM EDTA and 5 mM dithiothreitol) with 100 μM ATP, 400 μM dNTPs (each), 25 μM CTP, GTP, and UTP. As a positive control we measured the ssDNA-dependent ATPase activity of DnaB. We incubated 10 nM DnaB with 1 nM M13mp18 ssDNA (Guild Bioscience). As a negative control, we measured the ATPase activity during Pol III strand-displacement synthesis. This reaction does not include DnaB and would, therefore, detect only DnaB independent ATPase activity. The Pol III strand-displacement reaction contained 2 nM rolling-circle template, 30 nM αεθ, 10 nM τ_3_δδ’χψ, 30 nM β_2_, and 1 µM SSB_4_. Replication and unwinding reactions were set up in replication buffer and contained 2 nM rolling-circle template and. The unwinding reaction contained 10 nM DnaB_6_(DnaC)_6_. The replication reaction contained 10 nM DnaB_6_(DnaC)_6_, 30 nM αεθ, 10 nM τ_3_δδ’χψ, 30 nM β_2_, 75 nM DnaG, 1 µM SSB_4_. Reactions were incubated at 37 °C. Time points were taken at 0, 180, and 1800 s by quenching 20 μl of each reaction with 20 μl 1 × ADP Detection Mix (20 mM HEPES pH 7.5, 40 mM EDTA, 0.02% Brij-35, 4 nM ADP-AF633 tracer, 80 μg·ml^−1^ ADP^2^ antibody). After incubation at room temperature for 1 h, fluorescence polarization was measured using a POLARstar Omega (BMG Labtech) plate reader (584 nm ex, 602–622 nm em) using black 384-well plates (Greiner). A standard curve mimicking ATP hydrolysis was generated by measuring the fluorescence polarization of six concentrations of ADP in the presence of complementary concentrations of ATP to keep the total adenine nucleotide concentration constant (e.g., [ADP] + [ATP] = 100 μM). The measurements for the standard curve were carried out it the same buffer and quenched with 1 × ADP Detection Mix. A linear fit to the standard curve allowed conversion of polarization units to ADP concentration (Supplementary Fig. 8b).

To confirm that synthesis of DNA occurred, the replication and Pol III strand-displacement synthesis reactions were run on a 1% agarose gel in TAE buffer (40 mM Tris pH 8, 20 mM acetic acid, 1 mM EDTA), and stained with SYBR-Gold (Invitrogen) (Supplementary Fig. 8d, e). The efficiency of replication was calculated by measuring the integrated intensity of the template band and found to be 60%.

The expected amount of ADP generated during replication was calculated by assuming DnaB unwinds 2 nt for every ATP hydrolysed^9,11,58^, a new clamp is loaded for every Okazaki fragment, a replication rate of 500 bp s^−1^ (Fig. 1e), and a 60% replication efficiency (Supplementary Fig. 8d). For the expected amount of ADP generated during unwinding, we assumed an unwinding rate of 60 nt/s. For the expected amount of ADP in the Pol III strand-displacement assay we assumed ATP-dependent loading of a β_2_ clamp every 300 nt^58^, and a replication rate of 150 nt·s^−1^.

### DNA unwinding assays

To make the forked DNA substrate, 20 µM of a 3′ biotinylated oligonucleotide was annealed to 20 µM of a complementary 5′ Alexa Fluor 647 (AF647)-labelled oligo, by incubating at 65 °C for 5 min and cooling down to room temperature at 1 °C per minute^80,81^. The final products were stored at –80 °C.

5 pM of the DNA substrate was loaded onto the microfluidic flow cell at a rate of 10 μl/min until an appropriate surface density was achieved. To remove excess DNA molecules from solution the flow cell was washed with 200 μl replication buffer at a rate of 70 μl/min. 20 nM helicase was loaded in replication buffer in the presence of 100 μM ATP and incubated for 10 min. The reaction was initiated by loading 5 mM of the specified nucleotide in replication buffer at 30 °C for DnaB and 37 °C for CMG. Unwinding was imaged every 10 min for 1 h, with a laser power of 80 μW cm^−2^ an exposure time of 400 ms, with a constant buffer flow of 2 μl/min.

The time courses were fitted with a single exponential. The resulting parameters are reported in Supplementary Table 2.

### Expression and purification of human CMG

hCMG^82^, were expressed and purified from insect cells using the GoldenBac expression system^83^. Tni cells were infected at a density of 1 × 10^6^ cells/mL and incubated for 68 h post infection.

The cell pellet obtained from 2 L of Tni cells was resuspended in 120 mL of hCMG lysis buffer (40 mM HEPES-KOH pH 7.6, 0.005% Tween 20, 10% glycerol, 150 mM sodium acetate) supplemented with protease inhibitor cocktail (Roche). Once resuspended, cells were lysed via sonication and the lysate was clarified by centrifugation at 45,000 rpm (Ti45 rotor) for 45 min. The soluble fraction was incubated with 3 mL of pre-equilibrated M2 agarose anti-FLAG beads at 4 °C for 2 h. The beads were washed with 20 CV of hCMG lysis buffer. Bead-bound protein was eluted with 4 CV of hCMG lysis buffer supplemented with 0.5 mg/mL FLAG peptide via gravity flow FLAG peptide with 15 min incubation between each elution step. FLAG eluates were pooled and incubated with 0.5 mL Strep-Tactin Superflow beads at 4 °C for 1 h. The beads were washed with 10 CV of lysis buffer, followed by 10 CV of lysis buffer supplemented with 0.5 mM ATP and 5 mM magnesium acetate and the bound proteins were eluted with 10 × 1 CV of hCMG lysis buffer supplemented with 50 mM biotin. Peak fractions containing protein were pooled and fractionated on MonoQ 5/50 column with a salt gradient of 150–1000 mM KCl over 30 CV. Peak fractions from the MonoQ step were pooled and dialysed against CMG storage buffer (40 mM HEPES-KOH pH 7.6, 0.005% Tween 20, 10% glycerol, 80 mM potassium acetate, 1 mM DTT, and 2 mM Magnesium acetate).

### Statistics and reproducibility

The number of molecules or events analysed is indicated in the text or figure legends. Errors reported in this study represent the standard error of the mean (SEM) or the error of the fit, as indicated in the text or figure legends. Every single-molecule replication experiment was carried out at least in triplicate.

Differences between conditions were assessed using two-tailed Welch’s t-tests on the underlying distributions or replicate means as appropriate. The resulting *p*-values are reported in the figure legends, and statistically significant differences are indicated in the figures.

### Reporting summary

Further information on research design is available in the Nature Portfolio Reporting Summary linked to this article.

## Supplementary information

Supplementary Information

Reporting Summary

Transparent Peer Review file

## Data Availability

Single-molecule and LC-MS data have been deposited at Zenodo (10.5281/zenodo.15679243)^84^. All original code has been deposited on GitHub (https://github.com/Single-molecule-Biophysics-UOW).

## Code availability

All original code has been deposited on GitHub (https://github.com/Single-molecule-Biophysics-UOW).
