# Supplementary material for: ATP is dispensable for E. coli DNA replication and eukaryotic helicase activity
Source: Nat Commun. 2026 Jun 4;17:5358. doi: 10.1038/s41467-026-73893-5 (PMC13276069; doi:10.1038/s41467-026-73893-5)
Supplement: Supplementary file 1 — Supplementary Information [file 41467_2026_73893_MOESM1_ESM.pdf]

Supplemental Information

ATP is dispensable for *E. coli* DNA replication and eukaryotic helicase activity

Richard R. Spinks[1,4], Aleksa Lakic[1,4], Celine Kelso[1], Slobodan Jergic[1], Olga Yurieva[2], Zhi-Qiang Xu[1], Michael E. O'Donnell[2,3], Nicholas E. Dixon[1], Antoine M. van Oijen[1,*], Jacob S. Lewis[1*] & Lisanne M. Spenkelink[1,*]

[1]Molecular Horizons and School of Science, University of Wollongong, Wollongong, New South Wales, Australia.

[2]Laboratory of DNA Replication, Rockefeller University, New York, NY 10065, USA

[3]Howard Hughes Medical Institute, Cambridge, MA 02138, USA

[4]These authors contributed equally

*Correspondence to: vanoijen@uow.edu.au, jacobl@uow.edu.au, lisanne@uow.edu.au.

This file contains Supplementary Figs. 1–9 and Supplementary Tables 1–2.

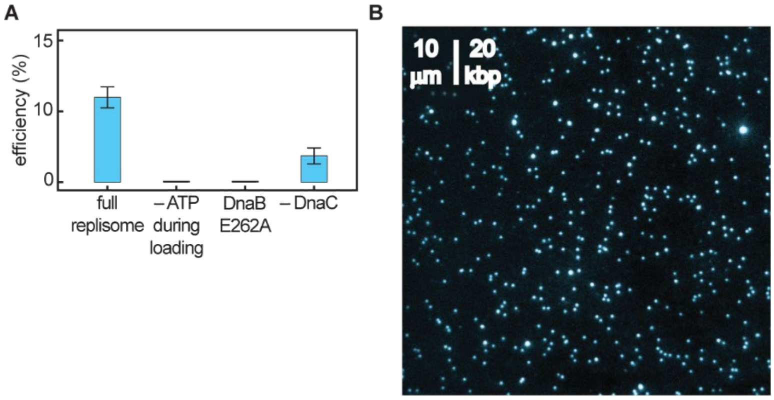

Supplementary Fig. 1 ATP is required for replication-competent loading of DnaB.

(A) Replication efficiencies for leading- and lagging-strand synthesis (same as in Fig. 1c), synthesis in the absence of ATP during DnaB loading, synthesis with DnaB-E262A in presence of ATP, and synthesis in the absence of DnaC during DnaB loading. (B) Representative field of view showing the lack of replication products in the leading and lagging-strand synthesis assay after omitting ATP from the DnaB loading phase. The image was recorded 2 min after replication initiation, where if the helicase was loaded in a manner that supports replication, we would expect to see long, replicating DNA products (Fig. 1c and Supplementary Data Fig. 2) instead of these un-replicated DNA templates.

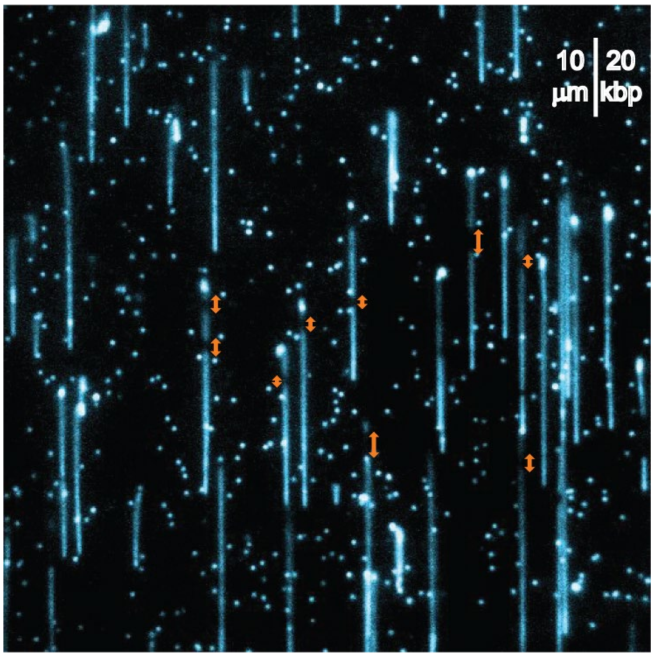

Supplementary Data Fig. 2 Inefficient priming in the absence of rNTPs.

Representative field of view of simultaneous leading- and lagging-strand replication in the absence of ATP (and all other rNTPs). Arrows indicate sporadic ssDNA gaps in the replication products as a result of inefficient priming of lagging-strand Okazaki fragments by DnaG primase using dNTPs.

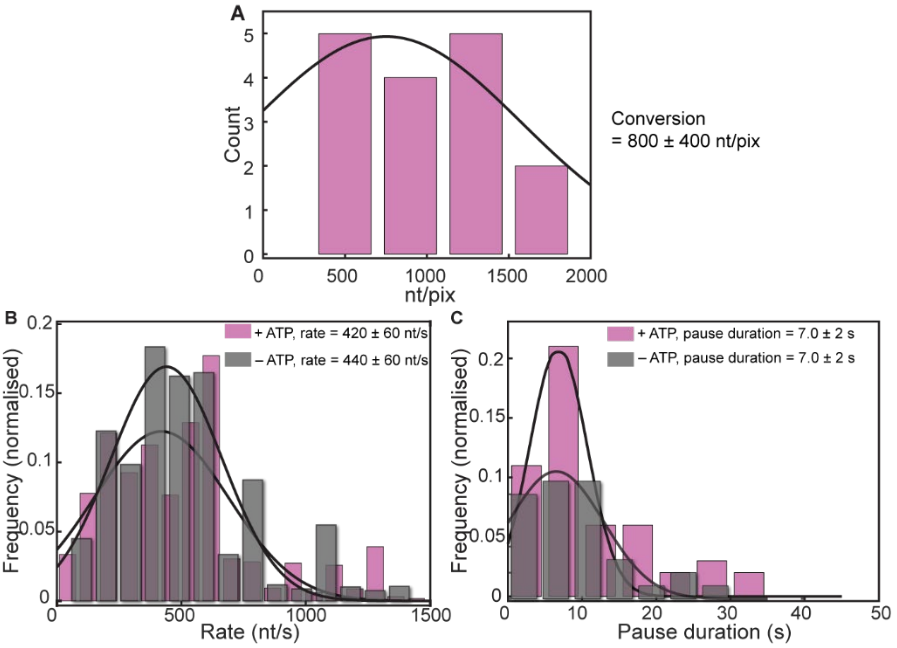

Supplementary Data Fig. 3 Leading-strand synthesis rates do not depend on the presence of ATP.

(A) Calibration histogram for quantification of the number of nucleotides per pixel of SSB-coated ssDNA in leading-strand synthesis (*N* = 16 molecules). The total fluorescence intensity of the SSB-coated DNA is divided by the intensity of a single SSB molecule to determine the number of bound SSB molecules. Using the known ssDNA footprint of SSB, this yields the total number of nucleotides in the product, which is then divided by the measured product length in pixels to obtain the nucleotide-per-pixel conversion factor. (B) Rate histograms of leading-strand replication in the presence (purple, *N* = 80 molecules, 51 pauses) and absence (grey, *N* = 116 molecules, 60 pauses) of ATP. (C) Histograms of the pause duration in the presence (purple, *N* = 80 molecules) and absence (grey, *N* = 116 molecules). Errors represent s.e.m. –

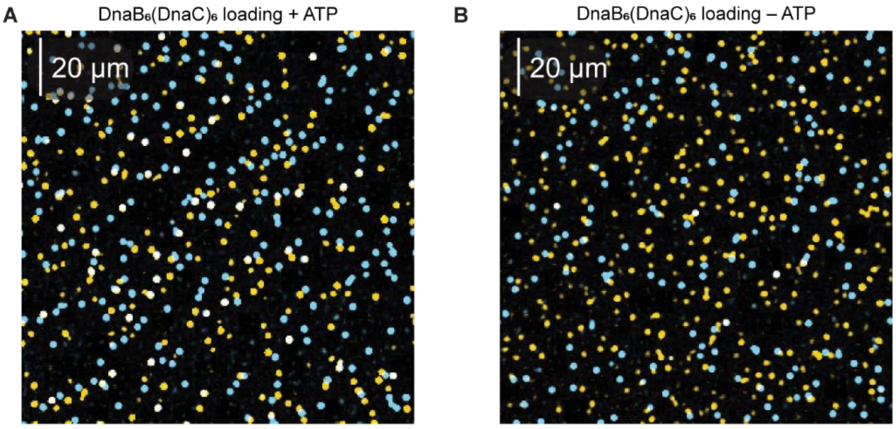

A

	# molecules	Colocalised	Chance
DNA	2067	17.1	4.2
DnaB	1372	25.8	2.8

B

	# molecules	Colocalised	Chance
DNA	1862	3.8	3.8
DnaB	2468	2.8	5.0

Supplementary Data Fig. 4 Effect of ATP on DnaB helicase loading.

To measure the DnaB loading efficiency we measured the colocalisation between DNA spots (blue) and fluorescently-labelled DnaB spots (orange) as a percentage of the total number of spots. (A) Typical field of view in the presence of ATP. Colocalised spots appear as white. The

table shows the total number of molecules analysed, colocalisation (%) and colocalisation by chance (%). (B) Typical field of view in the absence of ATP. Successfully loaded events would appear as white. The table depicts the total number of molecules analysed, colocalisation (%) and colocalisation by chance (%).

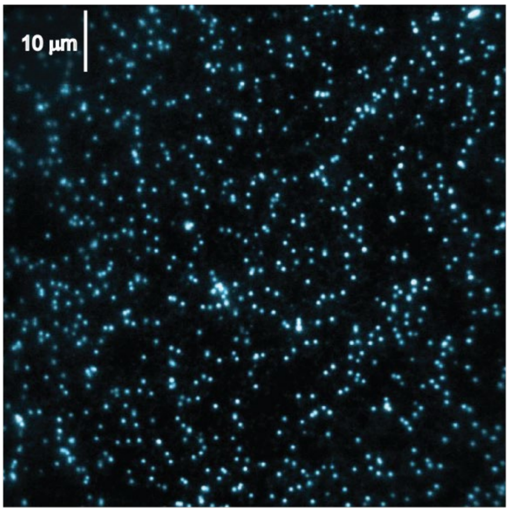

Supplementary Data Fig. 5 No replication products when DnaB is omitted.

Representative field of view demonstrating the lack of replication when DnaB is omitted from the leading-strand synthesis reaction. The image was taken 2 min after replication initiation. In contrast to Fig. 1c and Supplementary Data Fig. 3, no replication products were observed in the absence of DnaB. Strand-displacement synthesis is possible by the Pol III holoenzyme and SSB, but only at much higher concentration of dNTPs[44].

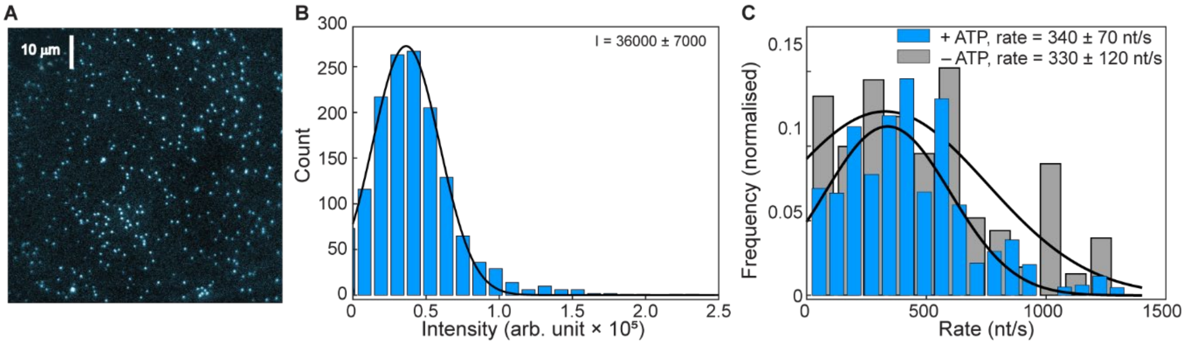

Supplementary Data Fig. 6 Minimal assay quantification.

(A) Typical field of view showing M13 ssDNA stained with SYTOX. (B) Histogram of the intensity of SYTOX-stained M13 ssDNA molecules. The black line represents a Gaussian fit to the data. The mean fluorescence intensity was divided by the known length of M13mp18 (7249 nt) to obtain the conversion factor between fluorescence intensity and nucleotide number, yielding 5 ± 1 arbitrary units nt^{-1}. Error represents s.e.m. (C) Histograms showing the rate of replication by the minimal replisome in the presence (blue) and absence (grey) of ATP. The black lines represent Gaussian fits to the data. Errors represent s.e.m.

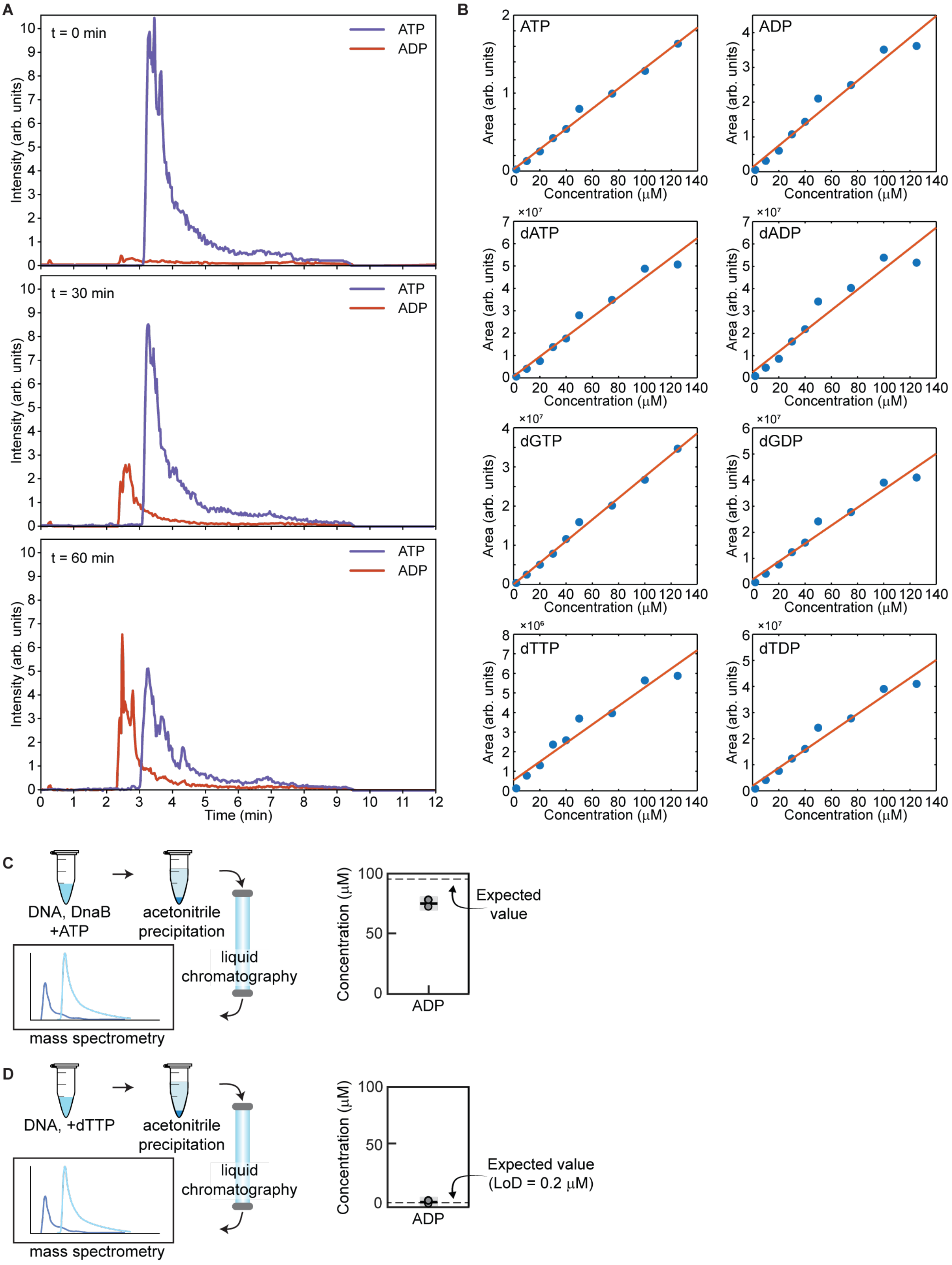

Supplementary Data Fig. 7 LC-MS ATPase control.

(a) Example LC-MS spectra showing the measurement of ATP and ADP at t = 0 (top), t = 30 min (middle), and t = 60 min (bottom). (b)Standard curves showing the relationship between nucleotide concentration and the area under the LC-MS curves. (c) Schematic of the LC-MS assay (left). 30nM DnaB is mixed with 10nM ssDNA and 100μM ATP. After 30 min the reaction is quenched with acetonitrile and the presence of ADP is quantified via LC-MS (see methods).

The measured concentration of ADP is close to the expected value (right). (d) Schematic of the LC-MS assay (left). DnaB is mixed with ssDNA and dTTP. After 30 min the reaction is quenched with acetonitrile and the presence of ADP is quantified via LC-MS (see methods). The measured concentration of ADP is at the limit of detection (LoD; right).

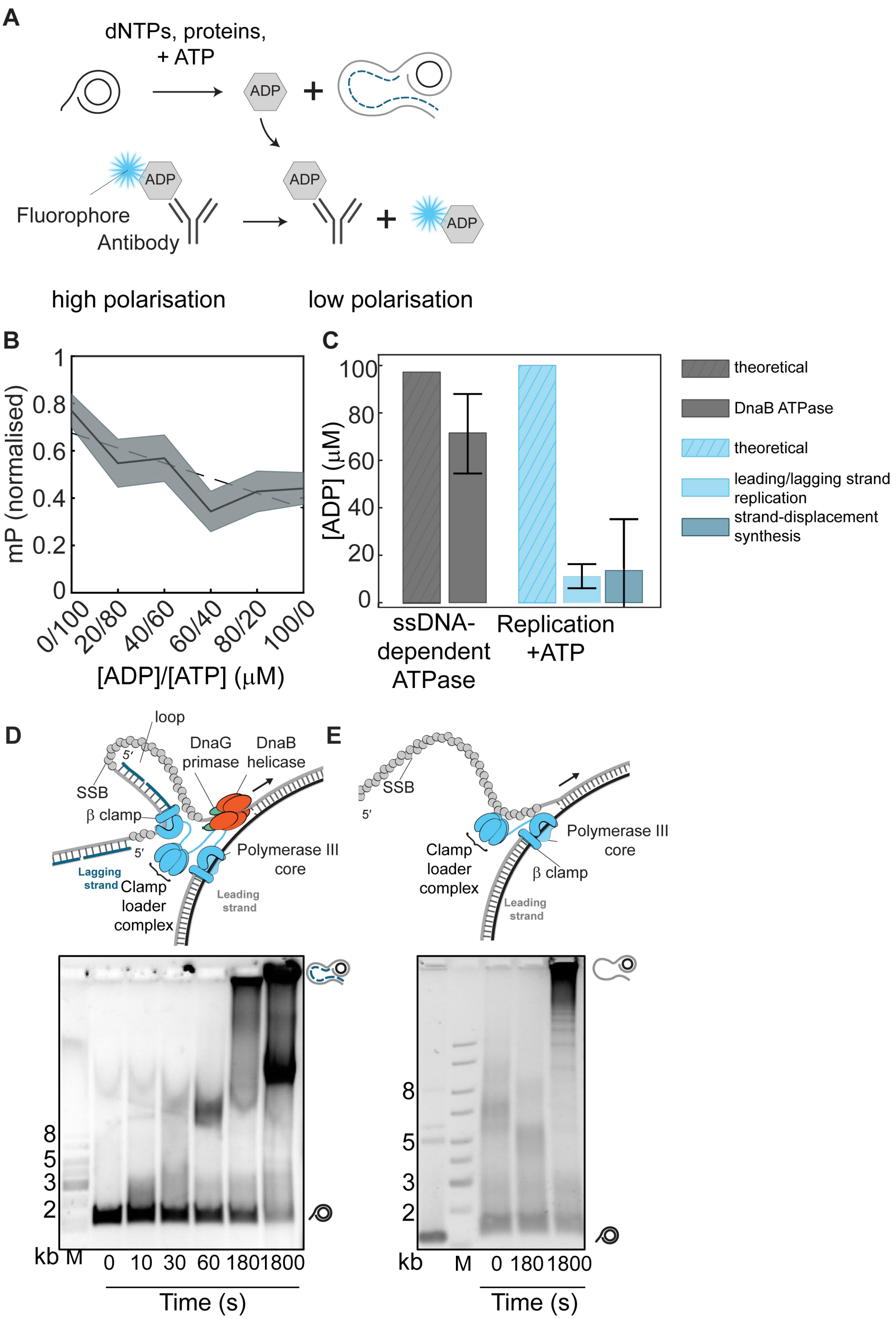
A
dNTPs, proteins, + ATP
ADP
Fluorophore
Antibody
high polarisation
low polarisation
B
mP (normalised)
[ADP]/[ATP] (μM)
0/100
20/80
40/60
60/40
80/20
100/0
C
[ADP] (μM)
theoretical
DnaB ATPase
theoretical
leading/lagging strand replication
strand-displacement synthesis
ssDNA-dependent ATPase
Replication +ATP
D
loop
DnaG primase
DnaB helicase
SSB
β clamp
5′
Lagging strand
Clamp loader complex
Leading strand
Polymerase III core
kb M
0 10 30 60 180 1800
Time (s)
E
SSB
5′
Polymerase III core
Clamp loader complex
β clamp
Leading strand
kb
M
0 180 1800
Time (s)

Supplementary Data Fig. 8 Fluorescence-polarisation measurement of ATPase activity during replication. (A) Schematic of the fluorescence polarisation ATPase assay. Fluorescent ADP is bound to an antibody. ADP generated by ATP hydrolysis displaces the fluorescent ADP of the antibody, resulting in a decrease in fluorescence polarisation. (B) Standard curve used to convert fluorescence (in mP) to ADP concentration. The shaded area represents the s.e.m. of three replicates. (C) Concentration of ADP measured after 30 minutes for ssDNA-dependent ATPase activity by DnaB (grey), and simultaneous leading- and lagging-strand replication (blue). (D) top, schematic representation of the replisome during leading-/lagging-strand synthesis. bottom, agarose gel showing DNA replication products from the same leading-/lagging-strand replication reaction that was used for measurement of ATPase activity. Measurement of the integrated intensity of the template band shows that 60% of the template was replicated. (E) top, schematic representation of Pol III strand-displacement, whereby DNA strand separation is achieved through polymerase activity of the Pol III HE, coupled to SSB on the displaced strand. bottom, agarose gel showing DNA replication products from the Pol III strand-displacement reaction that was used for measurement of ATPase activity.

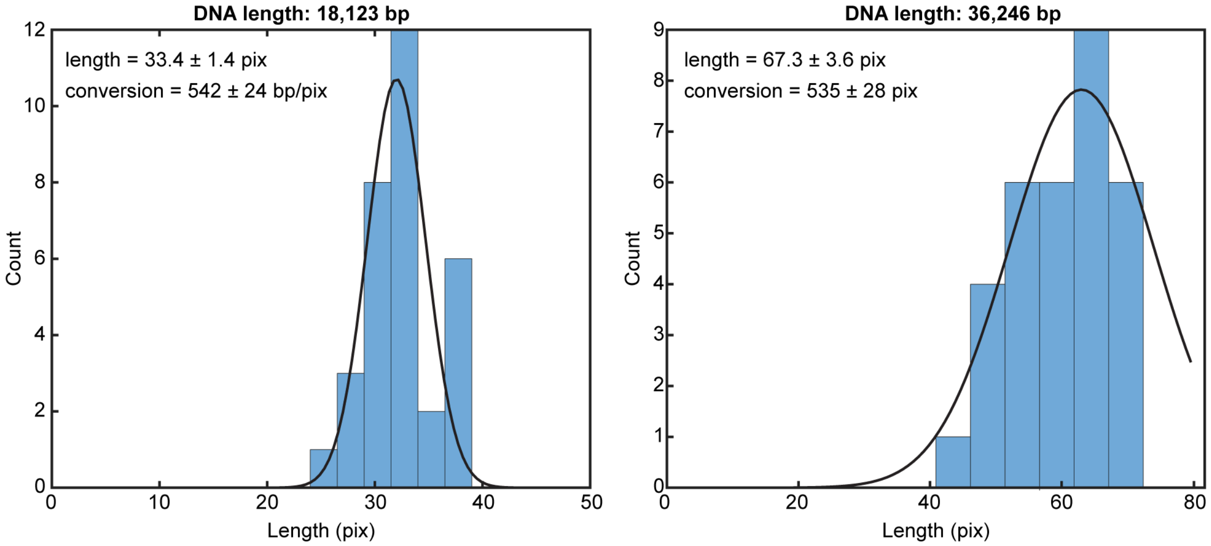

Supplementary Data Fig. 9 dsDNA length conversion. Histogram of the measured extension (pixels) of DNA substrates of known length under assay flow conditions. The black line represents a Gaussian fit to the data. The mean extension was used to calculate the base pairs per pixel (bp/pixel) conversion factor.

Supplementary Data Table 1. Optimised parameters for the declustering potential (DP), collision cell entrance and exit potential (EP and CXP, respectively) and collision energy (CE) for each compound.

Compound	Q1 *m/z*	Q3 *m/z*	DP (V)	EP (V)	CE (V)	CXP (V)
ATP	508.0	410.0	125	10	31	40
dATP	492.0	136.1	100	10	30	40
ADP	428.0	136.1	55	10	28	35
dADP	412.0	136.0	120	10	30	32
dGTP	508.0	152.0	1000	10	30	40
dGDP	428.0	152.1	70	10	22	35
TTP	483.0	81.1	145	10	16	37

dTDP	403.0	207.1	130	10	12	35

Supplementary Data Table 2. Fit parameters for the unwinding data in Fig. 5.

	nucleotide	A (% unwound)	k (min^{-1})
**DnaB**	ATP	34 ± 2	0.08 ± 0.02
	dTTP	23 ± 5	0.04 ± 0.02
**yCMG**	ATP	56 ± 9	0.04 ± 0.02
	dTTP	27.1 ± 0.7	0.048 ± 0.004
**hCMG**	ATP	33 ± 5	0.05 ± 0.02
	dTTP	28 ± 3	0.06 ± 0.02
