# Supplementary material for: ATP is dispensable for E. coli DNA replication and eukaryotic helicase activity
Source: Nat Commun. 2026 Jun 4;17:5358. doi: 10.1038/s41467-026-73893-5 (PMC13276069; doi:10.1038/s41467-026-73893-5)
Supplement: Supplementary file 3 — Transparent Peer Review file [file 41467_2026_73893_MOESM3_ESM.pdf]

nature portfolio

Peer Review File

ATP is dispensable for *E. coli* DNA replication and eukaryotic helicase activity

Corresponding Author: Dr Lisanne Spenkelink

This file contains all reviewer reports in order by version, followed by all author rebuttals in order by version.

Version 0:

Reviewer comments:

Reviewer #1

(Remarks to the Author)
In this manuscript, Spinks and Lakic et al. used single-molecule fluorescence imaging and mass spectrometry to investigate the chemical energy source of bacterial DNA replication machinery. They demonstrated that ATP is dispensable for full and leading strand replication and dTTP can be used as the energy source of E. coli replisome. The authors further demonstrated that nucleotide promiscuity is also present in human and yeast replicative helicase unwinding.
The measurements were carefully designed and carried out. This study is interesting to the biophysics and DNA replication communities. However, the manuscript lacks certain statistical analyses and control measurements. Some of the claims in this manuscript were not supported by the experimental results, and certain parts of the manuscript lack clarity. I recommend reconsidering the manuscript after the authors address the following comments.

Major points:
1. When the authors make claims by comparing two datasets, they should use statistical tests in addition to simply showing numbers and bar plots. Particularly, in Figs. 2 and 3, Extended data Figs. 3 and 6 and related main text, the (lack of) impact of ATP on loading efficiency, replication efficiency and replisome velocity should be supported by statistical tests showing if the datasets collected in the presence and absence of ATP are significantly different or not.
2. The authors made claims that are not supported by the data. Specifically:
2.1 The authors claimed that ‘DnaB also preferentially uses dTTP when unwinding DNA outside the context of the replisome’. This claim is not supported by the data presented in this manuscript. Fig. 5c actually shows the unwinding efficiency of DnaB is higher in the presence of ATP compared to dTTP.
2.2 The authors claimed in the title of Fig. 3 that “the polymerase is the main driving force of the replisome”. However, the data presented in this manuscript does not support this claim, but instead leads to the claim that hydrolysis of dTTP by DnaB is the main driving force of the replisome.
3. The authors’ results lead to the conclusion that the E. coli replisome predominantly uses dTTP over ATP. This conclusion raises important questions that were not addressed in the manuscript, specifically:
3.1 This manuscript shows DnaB unwinding efficiency is slightly higher in ATP (Fig. 5c). The related manuscript by Rashid and Pangeni et al. shows that ssDNA translocation velocity of DnaB in ATP and dTTP are similar, and that dTTP binds DnaB less efficiently than ATP. With these results in mind, it is quite surprising that the E. coli replisome predominantly uses dTTP, even in the presence of ATP. Could the authors discuss possible molecular mechanisms of this preference towards dTTP?
3.2 In E. coli cells, the in vivo concentration of dTTP is ~10 fold lower than ATP. It therefore seems less efficient to use dTTP as the main energy source for DNA replication, and this might increase the risk of dTTP depletion. Could the authors elaborate more on the connection between their results and the in vivo scenario?
4. The mass spectrometry assay in Fig. 4 lacks negative control in the absence of replication. The authors could omit DnaB or the DNA substrate to demonstrate that the generation of dTDP is coupled to DNA replication.
5. I encourage the authors to rule out possible ATP contamination in their assays related to nucleotide promiscuity to strengthen their claims, referring to an article previously published by one of the coauthors: N.Y. Yao, D. Zhang, O. Yurieva, & M.E. O’Donnell, CMG helicase can use ATPγS to unwind DNA: Implications for the rate-limiting step in the reaction mechanism, Proc. Natl. Acad. Sci. U.S.A. 119 (4) e2119580119, https://doi.org/10.1073/pnas.2119580119 (2022).

Minor points:
1. In Extended Data Fig. 4, a large fraction of DnaB fluorescent spots (orange) did not colocalize with the DNA template

(blue) even in the presence of ATP. Could the authors explain the reason behind these isolated DnaB spots, could it be non-specific adhesion of DnaB on the surface?
2. Extended Fig. 8c is poorly labeled. It is not clear what each bar refers to.
3. The figure panel labels (a, b, c…) are too small and hard to see.

Reviewer #2

(Remarks to the Author)
I co-reviewed this manuscript with one of the reviewers who provided the listed reports. This is part of the Nature Communications initiative to facilitate training in peer review and to provide appropriate recognition for Early Career Researchers who co-review manuscripts.

Reviewer #3

(Remarks to the Author)
Review of “ATP is dispensable for E. coli DNA replication and eukaryotic helicase 1 activity” for NAR. In this manuscript, the authors primarily utilize single molecule experiments complemented with bulk analysis to show that the DnaB helicase within the E. coli replisome does not require ATP for robust activity. Instead, they conclude the dTTP is utilized preferably even to ATP. The also show a similar dTTP utilization for the yeast CMG helicase which is of a different evolutionary type than DnaB. This work in interesting as for decades, ATP has been thought to be the primary fuel for DnaB unwinding. Instead, it would seem that ATP is needed for DnaB loading by DnaC but after release, DnaB can utilize almost any nucleotide to power unwinding. The work is high quality, however there are several instances where the story is incomplete or experimental information missing.

1) Figure 2c – Is there a significant difference +/- ATP with the full replisome? Adding p-values may help or labelling as n.s. if not. Add the same n.s. in Fig 3d.
2) Extended data Fig3. Regarding pausing. Is there any correlation of pausing with extended length such that pausing is more frequent at a specific sequence? Or is it just stochastic?
3) Page 5, line 175, the heading is confusing. “No ATP hydrolysis in the presence of ATP”
4) Page 5, line 180, you state that ATPase activity was performed at physiological concentrations of ATP, however, it would be helpful to include these concentrations in the Fig 4 legend. [ATP] and other nucleotide concentrations.
5) Fig. 4. According to M&M dCTP was also added to this reaction, but the data is not shown in the results. Why or can you include it for completeness?
6) Also Figure 4, If I am reading M&M correctly, there is 1.25 microM ATP and 400 nM of each dNTP. Typical dNTP concentrations in E. coli are around 100 microM or less, so your experimental concentration is 4x higher. Can this account for the increased utilization of dTTP in your assays? Goes to the argument of Km which is not discussed at all in this manuscript.
7) Figure 5, similar issues exist here in that experimental concentrations of dTTP (5 mM) are vastly higher than the physiological concentrations. Its one thing to say that DnaB can unwind DNA with dTTP, but it is another to say whether it does in the cell.
8) Figure 5, these are time courses; shouldn’t they be fit with an exponential curve to get better information on rate and product amount?
9) Discussion, second paragraph, lines 237-250, turns into a discussion on the cooperativity between DnaB unwinding and Pol III synthesis which is not the focal point of this manuscript. This should be scrapped and rewritten to better discuss the impact of the results from this manuscript on the utilization of dTTP as a primary fuel for DnaB and work in an analysis on whether the cellular concentrations of ATP and dNTPs would support DnaB unwinding in the cell based on Km and known concentrations of nucleotides.

Minor Comments
1) Page 9, line 351, correct CTp
2) Page 10, DNA unwinding assays. What temperature where they performed at?
3) There is no information on the purification of the yeast CMG?
4) Extended data Fig 7 legend. Concentrations for DnaB, ssDNA, and ATP should be included.
5) Extended data Figure 8. There is no information on how the fluorescence polarization assays was performed in M&M.

Reviewer #4

(Remarks to the Author)
In this paper, Spinks et al. report the unexpected finding that E. coli replisome elongation does not require ATP in a single-molecule rolling-circle DNA-replication assay contrary to the long-held view that ATP hydrolysis is essential for DNA unwinding. They further show that dTTP, rather than ATP, was preferentially hydrolyzed by DnaB during DNA replication via LC-MS assay. They also demonstrated that replicative helicases from other species (yeast CMG and human CMG) can also utilize dTTP for DNA unwinding. These results challenge the prevailing assumption that ATP hydrolysis is the primary energy source for helicase-driven DNA unwinding during replication. Based on these observations, the author proposed a “flex-fuel” property of replicative helicase, suggesting the cells may gain operational flexibility under varying cellular conditions. This work reframes our understanding of nucleotide fuels in the fundamental process of DNA replication.

While the central finding is intriguing, I have significant concerns regarding the experimental support and data presentation. Several figures lack sufficient details, and many statements are not described with enough clarity to allow full evaluation. The current manuscript requires major revision, including more comprehensive data presentation, rigorous quantification analysis, and incorporation of critical control experiments. The following issues should be addressed before the manuscript can be considered for publication.

1. Lines 81-90: "When we omit the helicase loader DnaC from the DnaB-loading step, the replication efficiency is reduced ~3-fold (Extended Data Fig. 1a). However, omitting ATP from the DnaB-loading step resulted in a complete lack of replication (Extended Data Fig.1a,b)."

The replication efficiency and DnaB loading efficiency are distinct concepts and should be clearly clarified. Based on Extended Data Fig. 4, both DnaC and ATP primarily affect DnaB loading. The author should reorganize or reconcile Extended Data Fig. 1 and 4, as well as other figures (e.g., Fig.2c, Fig. 3d), to provide a consistent definition and usage of "replication efficiency" , which will improve clarity for readers. By the way, in Extended Data Fig. 4, what's the difference between "colocalisation (%) and colocalisation by chance (%)"? The author should explain their meanings and how these metrics relate to DnaB loading ability.

2. In this manuscript, the author used multiple readouts to quantify DNA replication product length/replication rates under different conditions in single-molecule assays, but the calibration procedure seems very coarse.

In Fig. 1d and e, DNA product length appears to be measured with a dsDNA fluorescent stain; in Fig. 2 and Extended Data Fig. 3, by the intensity of fluorescently labeled SSB; and in Fig. 3 and Extended Data Fig. 6, by a ssDNA fluorescent stain. Extended Data Fig. 3A doesn't clearly explain how the "nucleotides per pixel" calibration for SSB-coated ssDNA was obtained. Similarly, Extended Data Fig. 6B does not explain how the conversion factor was derived. Using a single known ssDNA length for calibration is insufficient. The author should perform more thorough calibrations (more standards spanning the relevant dynamic range, replicate measurements, linearity/precision checks) to ensure robust correspondence between DNA length and fluorescence intensity across all assay modalities, so that the reported replication rates are accurate and internally consistent.

3. The author assessed the ATP and dNTPs hydrolysis by quantifying ADP and dNDPs at the endpoint of replication reactions in LC-MS experiments, leading to the conclusion that dTTP is preferentially hydrolyzed during DNA replication (Fig. 4a and Extended Data Fig. 7). This is a central claim of this story, yet the data presentation is unclear.

In Fig. 4a and Extended Data Fig. 7, there are only schematic diagrams for the LC-MS results, lacking the underlying original data with spectra/peak identities, retention times. Please include representative raw data and annotate how different nucleotide products were distinguished and quantified. The Methods (Line 349-407) describe the experimental setup, but the manuscript does not present results for each control sample mentioned. The author should provide the corresponding control data (e.g., buffer-only, nucleotide-only, enzyme-only, no-DNA, etc…), and explain how concentrations were converted and normalized from LC-MS signals, including calibration curves, internal standards, and correction for matrix effects. It's better to perform protein concentration titrations to confirm that the nucleotide hydrolysis arises from replisome activity rather than spontaneous breakdown under reaction conditions or nucleotide contamination.
By the way, in Extended Data Fig. 7B, please define "expected value", and explain how it was derived, and show the calculation underlying this expectation.

4. Lines 185-191, the author corroborate their findings using a fluorescence polarization: "We supported these findings by repeating the measurements using a fluorescence polarisation assay (Extended Data Fig. 8). Again, we see that ATPase activity during replication is much lower than expected. In fact, this reaction did not hydrolyse significantly more ATP than a Pol III strand-displacement reaction in which DnaB is omitted (Extended Data Fig. 8). We confirmed that efficient DNA replication occurred in all replication experiments, by running the DNA products from these assays on an agarose gel (Extended Data Fig. 8d,e). Interestingly, we again see that dTTP is hydrolysed preferentially (Fig. 4c)."

First of all, is the fluorescence polarization assay a validated, quantitative standard for measuring ATPase activity or ADP production in this context? In Extended Data Fig. 8B, the standard curve does not appear linear across the relevant range. How were fluorescence readouts converted to ADP concentrations in Extended Data Fig. 8C? The author should validate the assay cautiously to confirm it indeed can report on the ADP amount quantitatively.

Secondly, in Extended Data Fig. 8C, the bar categories are insufficiently defined. The legend states that grey bar indicate ssDNA-dependent ATPase activity by DnaB and blue bar indicates simultaneous leading- and lagging-strand replication, but the exact reaction compositions are unclear. Please define "ssDNA-dependent ATPase by DnaB" explicitly. Does this reaction use the same DNA substrate as replication but omit Pol III and other components? A schematic for each condition would greatly aid interpretation.

Thirdly, please point to specific panels supporting the statements in Lines 186-191: "Again, we see that ATPase activity during replication is much lower than expected. In fact, this reaction did not hydrolyse significantly more ATP than a Pol III strand-displacement reaction in which DnaB is omitted (Extended Data Fig. 8)." It's difficult to identify which data support these claims.

Lastly, in Extended Data Fig. 8D and E, the author verified the synthesized DNA products in bulk by running agarose gel.

Without DnaB, can Pol III and SSB mediated strand-displacement reaction still synthesize nascent DNA? The gel bands suggest similar upward shifts. If so, this implies substantial replication without helicase-driven unwinding !? Please clarify this result and explain the product identities.

5. In Lines 202-208, the author set up single-molecule unwinding assay to assess DnaB unwinding activity with ATP, dTTP, or AMPPNP. The author should add controls to verify that dsDNA lifetime were sufficiently long and not altered by nucleotide composition in the reaction buffer. In Fig. 5c, DnaB unwinds more slowly with dTTP than with ATP. How do the authors reconcile this with Fig. 4, which indicates preferential dTTP hydrolysis during DNA replication even when ATP is present?

6. In Lines 235-237: “Interestingly, the rate of unwinding (~1 nt/s) and the rate of strand displacement synthesis (~100 nt/s, Extended Data Fig. 8e) are both much smaller than the rate of DnaB-mediated replication (Fig. 1e, Extended Data Fig. 3b).” Please provide explicit methods and calculations for these rate estimates, including how ~1 nt/s and ~100 nt/s were derived, with references to the exact panels and fits in correct figures.

7. In Lines 234-250, the author discussed the coupling between DnaB helicase and polymerase. Please clarify why this discussion appears at this point and how it relates to the central question of fuel choice (ATP vs. dTTP) during replication.

8. Throughout the manuscript, DnaB was preloaded in ATP (required for loading per Extended Data Fig. 4), followed by ATP washout and initiation replication in dTTP-containing buffers. What is the physiological relevance of preferential dTTP use during elongation, while need ATP for loading? The author should expand the discussion to this point.

9. The author could examine the structures of DnaB and other tested helicases regarding nucleotide binding-pocket permissiveness. Are there structural data supporting that the ATP-binding pocket can accommodate dTTP?

10. Lines 171-174 and 217-218 cited reference 48 (an online conference abstract) to support their conclusions. Given that this is not a peer-reviewed publication and provides limited experimental and results details, the author might clarify this and temper the claims.

Version 1:

Reviewer comments:

Reviewer #1

(Remarks to the Author)
The authors have sufficiently addressed most of my comments. I recommend accepting this manuscript for publication after the authors address these remaining minor comments:
1) The authors should explain the statistical tests carried out in the relevant figure captions and methods section as well, in addition to mentioning in the rebuttal letter.
2) Fig. 4c caption: the authors should mention the ATP concentration (1.25 mM) to be consistent with Fig. 4b caption.
3) I appreciate that the authors added discussions taking the in vivo concentrations of ATP and dTTP into account. The authors are correct that DnaB could use other nucleotides to drive its progression if dTTP levels drop. However, dTTP is obviously also essential for synthesizing nascent DNA, where dTTP depletion could lead to replication stress. I believe it is still one step too far to claim that dTTP hydrolysis is the main driving force of E. coli DNA replication in vivo, as suggested by the first sentence of Discussion. The in vivo scenario might be more complex and dynamic than that, considering the flex-fuel behavior of DnaB. I suggest that the authors temper down this claim.

Reviewer #2

(Remarks to the Author)
I co-reviewed this manuscript with one of the reviewers who provided the listed reports. This is part of the Nature Communications initiative to facilitate training in peer review and to provide appropriate recognition for Early Career Researchers who co-review manuscripts.

Reviewer #3

(Remarks to the Author)
This is a much improved manuscript with better statistical comparisons and integrated discussions on nucleotide utilization within the E. coli replisome. The authors have included additional experiment to adequately respond to all the reviewer comments.

Reviewer #4

(Remarks to the Author)
The authors have improved the clarity of the manuscript and addressed all of my questions. It is acceptable for publication.

Open Access This Peer Review File is licensed under a Creative Commons Attribution 4.0 International License, which permits use, sharing, adaptation, distribution and reproduction in any medium or format, as long as you give appropriate credit to the original author(s) and the source, provide a link to the Creative Commons license, and indicate if changes were made.
In cases where reviewers are anonymous, credit should be given to 'Anonymous Referee' and the source.
The images or other third party material in this Peer Review File are included in the article’s Creative Commons license, unless indicated otherwise in a credit line to the material. If material is not included in the article’s Creative Commons license and your intended use is not permitted by statutory regulation or exceeds the permitted use, you will need to obtain permission directly from the copyright holder.
To view a copy of this license, visit https://creativecommons.org/licenses/by/4.0/

We thank the reviewers for their helpful comments and suggestions. Based on their reviews, we have carried out extra experiments and data analysis. Please find a point-by-point response to the reviewers' comments below.

Reviewer #1 (Remarks to the Author):

In this manuscript, Spinks and Lakic et al. used single-molecule fluorescence imaging and mass spectrometry to investigate the chemical energy source of bacterial DNA replication machinery. They demonstrated that ATP is dispensable for full and leading strand replication and dTTP can be used as the energy source of E. coli replisome. The authors further demonstrated that nucleotide promiscuity is also present in human and yeast replicative helicase unwinding.

The measurements were carefully designed and carried out. This study is interesting to the biophysics and DNA replication communities. However, the manuscript lacks certain statistical analyses and control measurements. Some of the claims in this manuscript were not supported by the experimental results, and certain parts of the manuscript lack clarity. I recommend reconsidering the manuscript after the authors address the following comments.

Major points:

1. When the authors make claims by comparing two datasets, they should use statistical tests in addition to simply showing numbers and bar plots. Particularly, in Figs. 2 and 3, Extended data Figs. 3 and 6 and related main text, the (lack of) impact of ATP on loading efficiency, replication efficiency and replisome velocity should be supported by statistical tests showing if the datasets collected in the presence and absence of ATP are significantly different or not.

We have now performed statistical analyses for all comparisons between +ATP and –ATP conditions in Figs. 2 and 3 and Extended Data Figs. 3 and 6. Specifically, differences were assessed using two-tailed Welch's t-tests on the underlying distributions or replicate means as appropriate. The resulting p-values are reported in the figure legends, and statistically significant differences are indicated in the figures.

2. The authors made claims that are not supported by the data. Specifically:

2.1 The authors claimed that 'DnaB also preferentially uses dTTP when unwinding DNA outside the context of the replisome'. This claim is not supported by the data presented in this manuscript. Fig. 5c actually shows the unwinding efficiency of DnaB is higher in the presence of ATP compared to dTTP.

We have changed the text and removed the word 'preferentially' when referring to DnaB unwinding DNA outside of the context of the replisome.

2.2 The authors claimed in the title of Fig. 3 that "the polymerase is the main driving force of the replisome". However, the data presented in this manuscript does not support this claim, but instead leads to the claim that hydrolysis of dTTP by DnaB is the main driving force of the replisome.

We have changed the title of Fig 3 to: Minimal-replisome assay provides evidence that SSB binding does not contribute to unwinding.

3. The authors' results lead to the conclusion that the E. coli replisome predominantly uses dTTP over ATP. This conclusion raises important questions that were not addressed in the manuscript, specifically:

3.1 This manuscript shows DnaB unwinding efficiency is slightly higher in ATP (Fig. 5c). The related manuscript by Rashid and Pangeni et al. shows that ssDNA translocation velocity of DnaB in ATP and dTTP are similar, and that dTTP binds DnaB less efficiently than ATP. With these results in mind, it is quite surprising that the E. coli replisome predominantly uses dTTP, even in the presence of ATP. Could the authors discuss possible molecular mechanisms of this preference towards dTTP?

We agree that the preference for dTTP during replication is not explained by unwinding or translocation assays alone. A plausible explanation is that DnaB adopts distinct conformational states when translocating, when unwinding, and when engaged within the replisome, which may alter nucleotide usage. However, we have no direct evidence distinguishing between these possibilities and therefore do not want to speculate further. We have added a sentence to the Discussion noting that the mechanism underlying the dTTP preference remains unknown and will require future investigation. The text now reads: " Unwinding (Fig. 5c) and translocation assays[48] do not show the strong dTTP preference observed during replication, indicating that this is a replisome-engaged behaviour. The mechanism underlying this preference is currently unknown and will require future investigation."

3.2 In E. coli cells, the in vivo concentration of dTTP is ~10 fold lower than ATP. It therefore seems less efficient to use dTTP as the main energy source for DNA replication, and this might increase the risk of dTTP depletion. Could the authors elaborate more on the connection between their results and the in vivo scenario?

In our mass-spectrometry assays measuring the generation of dTDP and ADP in a replication reaction with ATP (Fig. 4b, middle) we have a ~3-fold excess of ATP. Under these circumstances we see ~16-fold more dTDP generated than ADP. Indeed ATP is ~10-fold more abundant than dTTP in the cell. If we assume a simple linear relationship, this corresponds to $\sim 16 \times (3/10) \approx 5$-fold greater utilization of dTTP than ATP in vivo. Importantly, DnaB is a flex-fuel motor and can use other r/dNTPs to sustain progression if dTTP levels drop. We have added the following text to the Discussion: "In our assays (Fig. 4b, middle) we have a 3-fold excess of ATP, yet dTTP is preferentially used by ~16-fold. In live cells ATP is in a ~10-fold excess over dTTP[58]. Assuming a simple linear relation between ATP/dTTP ratio and nucleotide usage, this still implies ~5-fold greater use of dTTP."

4. The mass spectrometry assay in Fig. 4 lacks negative control in the absence of replication. The authors could omit DnaB or the DNA substrate to demonstrate that the generation of dTDP is coupled to DNA replication.

We carried out the control reaction containing DNA and dTTP. Under these conditions, dTDP was not detected. These results have been added to Extended data Fig. 7 (panels C,D).

5. I encourage the authors to rule out possible ATP contamination in their assays related to nucleotide promiscuity to strengthen their claims, referring to an article previously published by one of the coauthors: N.Y. Yao, D. Zhang, O. Yurieva, & M.E. O'Donnell, CMG helicase can use ATPγS to unwind DNA: Implications for the rate-limiting step in the reaction mechanism, Proc. Natl. Acad. Sci. U.S.A. 119 (4) e2119580119, https://doi.org/10.1073/pnas.2119580119 (2022).

We note that in Fig. 4b, where the *E. coli* replication reaction is carried out without added ATP, no ADP is detected. If ATP contamination were present at functionally relevant levels, ADP would be observed in this condition. To rule out ATP contamination in the CMG preps, we carried out the unwinding assays in the absence of any nucleotide. These data showed no unwinding of the DNA substrate. The data have been added to Fig. 5.

Minor points:

1. In Extended Data Fig. 4, a large fraction of DnaB fluorescent spots (orange) did not colocalize with the DNA template (blue) even in the presence of ATP. Could the authors explain the reason behind these isolated DnaB spots, could it be non-specific adhesion of DnaB on the surface?

Indeed. Some fluorescently labelled DnaB molecules bind non-specifically to the surface, resulting in isolated DnaB spots that do not colocalise with the DNA. These contribute to a small background level of apparent colocalisation, which we account for as the "colocalisation by chance" value reported in the tables in the figure.

2. Extended Fig. 8c is poorly labeled. It is not clear what each bar refers to.

The labels in Extended Data Fig. 8c have now been clarified.

3. The figure panel labels (a, b, c…) are too small and hard to see.

The panel labels follow the journal's required formatting guidelines including font size.

Reviewer #2 (Remarks to the Author):

I co-reviewed this manuscript with one of the reviewers who provided the listed reports. This is part of the Nature Communications initiative to facilitate training in peer review and to provide appropriate recognition for Early Career Researchers who co-review manuscripts.

Reviewer #3 (Remarks to the Author):

Review of "ATP is dispensable for E. coli DNA replication and eukaryotic helicase 1 activity" for NAR. In this manuscript, the authors primarily utilize single molecule experiments complemented with bulk analysis to show that the DnaB helicase within the E. coli replisome does not require ATP for robust activity. Instead, they conclude the dTTP is utilized preferably even to ATP. The also show a similar dTTP utilization for the yeast CMG helicase which is of a different evolutionary type than DnaB. This work in interesting as for decades, ATP has been thought to be the primary fuel for DnaB unwinding. Instead, it would seem that ATP is needed for DnaB loading by DnaC but after release, DnaB can utilize almost any nucleotide to power unwinding. The work is high quality, however there are several instances where the story is incomplete or experimental information missing.

1) Figure 2c – Is there a significant difference +/- ATP with the full replisome? Adding p-values may help or labelling as n.s. if not. Add the same n.s. in Fig 3d.

See also our response to Reviewer 1, Comment 1. We have now added the statistical significance to Fig. 2c and labelled the comparison in Fig. 3d as n.s. where appropriate.

2) Extended data Fig3. Regarding pausing. Is there any correlation of pausing with extended length such that pausing is more frequent at a specific sequence? Or is it just stochastic?

We have examined this and did not find any correlation between pausing and position or sequence features within the template. Within the spatial and temporal resolution of our measurements, pausing appears to be stochastic.

3) Page 5, line 175, the heading is confusing. "No ATP hydrolysis in the presence of ATP"

We have changed the heading to: Even with ATP added it is not hydrolysed

4) Page 5, line 180, you state that ATPase activity was performed at physiological concentrations of ATP, however, it would be helpful to include these concentrations in the Fig 4 legend. [ATP] and other nucleotide concentrations.

We have added the concentration of ATP to the legend. To avoid a very lengthy caption, we refer the reader to the Methods section for the concentrations of the other nucleotides. The caption for Fig. 4b now reads: "Amount of ADP, dADP, and dTDP generated after 30 min of replication in the absence of 1.25 mM ATP (see methods for concentrations of other nucleotides)."

5) Fig. 4. According to M&M dCTP was also added to this reaction, but the data is not shown in the results. Why or can you include it for completeness?

The reference to dCTP in the Methods was an error. We have corrected the Methods accordingly.

6) Also Figure 4, If I am reading M&M correctly, there is 1.25 microM ATP and 400 nM of each dNTP. Typical dNTP concentrations in E. coli are around 100 microM or less, so your experimental concentration is 4x higher. Can this account for the increased utilization of dTTP in your assays? Goes to the argument of Km which is not discussed at all in this manuscript.

The intracellular concentrations of ATP and dNTPs in E. coli are approximately ~3 mM ATP and ~300 μM dTTP (Buckstein et al., 2007, J. Bacteriol.). These values are very similar to those used in our assays (1.25 mM ATP and 400 μM dTTP). See also our response to Reviewer 1, Comment 3.2.

7) Figure 5, similar issues exist here in that experimental concentrations of dTTP (5 mM) are vastly higher than the physiological concentrations. Its one thing to say that DnaB can unwind DNA with dTTP, but it is another to say whether it does in the cell.

The elevated dTTP concentration (5 mM) in Fig. 5 is consistent with standard practice in helicase unwinding assays (e.g. Jeong et al., 2004, PNAS, Lee & Richardson, 2010 JBC; Galletto et al., 2004, JMB, (15381422 and 15381423)) and is used to ensure that nucleotide supply is not rate-limiting. This allows us to specifically assess whether DnaB's unwinding mechanism is tolerant to nucleotide identity, rather than to recapitulate physiological conditions.

8) Figure 5, these are time courses; shouldn't they be fit with an exponential curve to get better information on rate and product amount?

We have added the fit parameters to Extended Data Table 2.

9) Discussion, second paragraph, lines 237-250, turns into a discussion on the cooperativity between DnaB unwinding and Pol III synthesis which is not the focal point of this manuscript. This should be scrapped and rewritten to better discuss the impact of the results from this manuscript on the utilization of dTTP as a primary fuel for DnaB and work in an analysis on whether the cellular concentrations of ATP and dNTPs would support DnaB unwinding in the cell based on Km and known concentrations of nucleotides.

We have revised this section of the Discussion to more clearly connect the cooperativity between DnaB and Pol III to the observed difference in nucleotide usage during replication versus unwinding/translocation. We have also added a short section linking our in vitro

nucleotide usage to intracellular nucleotide concentrations. See also our response to Reviewer 1, Comment 3.2.

Minor Comments

1) Page 9, line 351, correct CTp

CTP is now removed.

2) Page 10, DNA unwinding assays. What temperature where they performed at?

The assays were carried out at 30°C for DnaB and 37°C for CMG. We have now included this in the methods.

3) There is no information on the purification of the yeast CMG?

The yeast CMG was purified as described in Langston et al, 2014, PNAS. We have now added this reference under the 'Proteins' section in the Methods.

4) Extended data Fig 7 legend. Concentrations for DnaB, ssDNA, and ATP should be included.

We have now added these concentrations in the caption.

5) Extended data Figure 8. There is no information on how the fluorescence polarization assays was performed in M&M.

The method has been added.

Reviewer #4 (Remarks to the Author):

In this paper, Spinks et al. report the unexpected finding that E. coli replisome elongation does not require ATP in a single-molecule rolling-circle DNA-replication assay□contrary to the long-held view that ATP hydrolysis is essential for DNA unwinding. They further show that dTTP, rather than ATP, was preferentially hydrolyzed by DnaB during DNA replication via LC-MS assay. They also demonstrated that replicative helicases from other species (yeast CMG and human CMG) can also utilize dTTP for DNA unwinding. These results challenge the prevailing assumption that ATP hydrolysis is the primary energy source for helicase-driven DNA unwinding during replication. Based on these observations, the author proposed a "flex-fuel" property of replicative helicase, suggesting the cells may gain operational flexibility under varying cellular conditions. This work reframes our understanding of nucleotide fuels in the fundamental process of DNA replication.

While the central finding is intriguing, I have significant concerns regarding the experimental support and data presentation. Several figures lack sufficient details, and many statements are not described with enough clarity to allow full evaluation. The current manuscript requires major revision, including more comprehensive data presentation, rigorous quantification analysis, and incorporation of critical control experiments. The following issues should be addressed before the manuscript can be considered for publication.

1. Lines 81-90: "When we omit the helicase loader DnaC from the DnaB-loading step, the replication efficiency is reduced ~3-fold (Extended Data Fig. 1a). However, omitting ATP from the DnaB-loading step resulted in a complete lack of replication (Extended Data Fig.1a,b)."

The replication efficiency and DnaB loading efficiency are distinct concepts and should be clearly clarified. Based on Extended Data Fig. 4, both DnaC and ATP primarily affect DnaB

loading. The author should reorganize or reconcile Extended Data Fig. 1 and 4, as well as other figures (e.g., Fig.2c, Fig. 3d), to provide a consistent definition and usage of "replication efficiency" , which will improve clarity for readers. By the way, in Extended Data Fig. 4, what's the difference between "colocalisation (%) and colocalisation by chance (%)"? The author should explain their meanings and how these metrics relate to DnaB loading ability.

Replication efficiency is defined as the number of replication events divided by the number of DNA templates present at the start of the experiment. As such, this efficiency reflects multiple factors (inc DnaB loading efficiency, loading efficiencies of other replisome components, DNA template quality, etc).

We have added this definition to the text. The text now reads:

"When we omit the helicase loader DnaC from the DnaB-loading step, the replication efficiency (defined as the number of replication events over the number of DNA templates present at the start) is reduced ~3-fold…"

In Extended Data Fig. 4, the percentage colocalisation is equivalent to DnaB loading efficiency. To reflect this, we have updated the caption of Extended Data Fig. 4 to:

"Effect of ATP on DnaB helicase loading efficiency. To measure the DnaB loading efficiency we measured the colocalisation between DNA spots (blue) and fluorescently-labelled DnaB spots (orange) as a percentage of the total number of spots. (A) Typical field of view in the presence of ATP. Colocalised spots appear as white. The table shows the total number of molecules analysed, colocalisation (%) and colocalisation by chance (%). (B) Typical field of view in the absence of ATP. Successfully loaded events would appear as white. The table depicts the total number of molecules analysed, colocalisation (%) and colocalisation by chance (%).

Under these definitions, Extended Data Fig. 1, Extended Data Fig. 4, Fig. 2c, and Fig. 3d are consistent:

- Extended Data Fig. 1 shows that omitting ATP from the DnaB-loading step (with DnaC present) results in 0% replication efficiency, indicating that ATP is required for loading.
- Extended Data Fig. 4 directly shows that DnaB loading is lost in the absence of ATP, consistent with the result above.
- Fig. 2c shows that replication does not occur in the absence of DnaB, indicating that DnaB is essential for strand separation.
- Fig. 3d shows that replication can still occur when SSB is omitted (in the presence of DnaB, DnaC, and ATP during loading), indicating that SSB is not the driving factor for replication initiation.

As described in the methods, colocalisation by chance was calculated based on area overlap and reflects the % colocalisation that would be observed purely by chance, rather than through a physical interaction. This number (~5%) reflects the threshold below which we cannot measure colocalisation accurately.

2. In this manuscript, the author used multiple readouts to quantify DNA replication product length/replication rates under different conditions in single-molecule assays, but the calibration procedure seems very coarse.

In Fig. 1d and e, DNA product length appears to be measured with a dsDNA fluorescent stain; in Fig. 2 and Extended Data Fig. 3, by the intensity of fluorescently labeled SSB; and in Fig. 3 and Extended Data Fig. 6, by a ssDNA fluorescent stain. Extended Data Fig. 3A

doesn't clearly explain how the "nucleotides per pixel" calibration for SSB-coated ssDNA was obtained. Similarly, Extended Data Fig. 6B does not explain how the conversion factor was derived. Using a single known ssDNA length for calibration is insufficient. The author should perform more thorough calibrations (more standards spanning the relevant dynamic range, replicate measurements, linearity/precision checks) to ensure robust correspondence between DNA length and fluorescence intensity across all assay modalities, so that the reported replication rates are accurate and internally consistent.

To calibrate the conversion between DNA extension and nucleotide length, we measured the stretched length of two DNA substrates of known size (18,123 bp and 36,246 bp) under the same flow and imaging conditions used in the assay. The resulting conversion factors were consistent (542 ± 24 and 535 ± 28 bp/pixel), and the mean value was used to convert measured extension into nucleotide length. This approach follows common practice in single-molecule DNA replication assays (Tanner et al., 2009, NAR; Graham et al., 2017, Mol Cell; Spenkelink & Lewis et al., 2017, eLife). We have added these data to Extended Data Fig. 9.

For the SSB-based measurements (Fig. 2 and Extended Data Fig. 3), the nucleotide-per-pixel calibration is derived from the fluorescence intensity of SSB bound to the replication product. Specifically, the total fluorescence intensity of the SSB-coated DNA is divided by the intensity of a single SSB molecule to determine the number of bound SSB molecules. Using the known ssDNA footprint of SSB, this yields the total number of nucleotides in the product, which is then divided by the measured product length in pixels to obtain the nucleotide-per-pixel conversion factor. This procedure is now clarified in the legend of Extended Data Fig. 3A.

SYTOX Orange produces a robust ssDNA signal only when substantial amounts of ssDNA collapse into a compact blob. Short ssDNA molecules do not generate a sufficiently signal under. For this reason, we used M13mp18 ssDNA (7249 nt) as the calibration standard. Importantly, the ssDNA products generated in our replication assays are substantially longer than M13. We have clarified the calibration of the ssDNA length in Extended Data Fig. 6B. The caption now reads: "Histogram of the intensity of SYTOX-stained M13 ssDNA molecules. The black line represents a Gaussian fit to the data. The mean fluorescence intensity was divided by the known length of M13mp18 (7249 nt) to obtain the conversion factor between fluorescence intensity and nucleotide number, yielding 5 ± 1 arbitrary units nt^{-1}."

3. The author assessed the ATP and dNTPs hydrolysis by quantifying ADP and dNDPs at the endpoint of replication reactions in LC-MS experiments, leading to the conclusion that dTTP is preferentially hydrolyzed during DNA replication (Fig. 4a and Extended Data Fig. 7). This is a central claim of this story, yet the data presentation is unclear.

In Fig. 4a and Extended Data Fig. 7, there are only schematic diagrams for the LC-MS results, lacking the underlying original data with spectra/peak identities, retention times. Please include representative raw data and annotate how different nucleotide products were distinguished and quantified. The Methods (Line 349-407) describe the experimental setup, but the manuscript does not present results for each control sample mentioned. The author should provide the corresponding control data (e.g., buffer-only, nucleotide-only, enzyme-only, no-DNA, etc…), and explain how concentrations were converted and normalized from LC-MS signals, including calibration curves, internal standards, and correction for matrix effects. It's better to perform protein concentration titrations to confirm that the nucleotide

hydrolysis arises from replisome activity rather than spontaneous breakdown under reaction conditions or nucleotide contamination.

By the way, in Extended Data Fig. 7B, please define “expected value”, and explain how it was derived, and show the calculation underlying this expectation.

To clarify the LC–MS measurements, we have expanded Extended Data Fig. 7 and revised the accompanying description in the manuscript.

Representative LC–MS spectra have now been added to Extended Data Fig. 7, showing the detection of the nucleotide products and their corresponding retention times and peak assignments.

We have also included the no-nucleotide control reaction in Extended Data Fig. 7. Under these conditions no ADP was detected, indicating that the nucleotide hydrolysis signal arises from the replication reaction rather than background signal.

In addition, we have added the calibration curves (standard curves) used to convert LC–MS peak areas into nucleotide concentrations. These are now shown in Extended Data Fig. 7 and the Methods section clarifies how nucleotide concentrations were derived from the LC–MS signals.

Finally, the definition of the “expected value” in Extended Data Fig. 7b has been clarified. This value is calculated from the measured replication rate together with the literature value for ATP turnover per nucleotide incorporated, and the calculation is now described in the figure legend.

4. Lines 185-191, the author corroborate their findings using a fluorescence polarization: “We supported these findings by repeating the measurements using a fluorescence polarisation assay (Extended Data Fig. 8). Again, we see that ATPase activity during replication is much lower than expected. In fact, this reaction did not hydrolyse significantly more ATP than a Pol III strand-displacement reaction in which DnaB is omitted (Extended Data Fig. 8). We confirmed that efficient DNA replication occurred in all replication experiments, by running the DNA products from these assays on an agarose gel (Extended Data Fig. 8d,e). Interestingly, we again see that dTTP is hydrolysed preferentially (Fig. 4c).”

First of all, is the fluorescence polarization assay a validated, quantitative standard for measuring ATPase activity or ADP production in this context? In Extended Data Fig. 8B, the standard curve does not appear linear across the relevant range. How were fluorescence readouts converted to ADP concentrations in Extended Data Fig. 8C? The author should validate the assay cautiously to confirm it indeed can report on the ADP amount quantitatively.

We have now added a section to the Methods detailing the fluorescence polarisation (FP) assay used to quantify ADP production, including the generation of the ADP/ATP standard curve and the conversion of fluorescence polarisation values to ADP concentrations.

Secondly, in Extended Data Fig. 8C, the bar categories are insufficiently defined. The legend states that grey bar indicate ssDNA-dependent ATPase activity by DnaB and blue bar indicates simultaneous leading- and lagging-strand replication, but the exact reaction compositions are unclear. Please define “ssDNA-dependent ATPase by DnaB” explicitly. Does this reaction use the same DNA substrate as replication but omit Pol III and other components? A schematic for each condition would greatly aid interpretation.

We have clarified the composition of these reactions in the Methods and added a detailed legend to Extended Data Fig. 8C describing the components present in each condition.

Thirdly, please point to specific panels supporting the statements in Lines 186-191: “Again, we see that ATPase activity during replication is much lower than expected. In fact, this reaction did not hydrolyse significantly more ATP than a Pol III strand-displacement reaction in which DnaB is omitted (Extended Data Fig. 8).” It’s difficult to identify which data support these claims.

We have clarified the relevant figure panel references in the manuscript and now explicitly refer to Extended Data Fig. 8c and 8e where these data are shown.

Lastly, in Extended Data Fig. 8D and E, the author verified the synthesized DNA products in bulk by running agarose gel. Without DnaB, can Pol III and SSB mediated strand-displacement reaction still synthesize nascent DNA? The gel bands suggest similar upward shifts. If so, this implies substantial replication without helicase-driven unwinding !? Please clarify this result and explain the product identities.

Yes, the *E. coli* Pol III holoenzyme is known to carry out substantial DNA synthesis in the absence of a helicase through strand-displacement activity. This behaviour has been well documented (Canceill & Ehrlich, 1996, PNAS; Stephens & McMacken, 1997, JBC; Yao et al., 2000, JBC; Xu & Marians, 2003, Mol. Cell; Yuan & McHenry, 2009, JBC). We included this reaction as a control because strand-displacement synthesis by Pol III is ATP-independent. The identity of the product is clarified by the icons next to the gel (Extended Data Fig. 8E)

5. In Lines 202-208, the author set up single-molecule unwinding assay to assess DnaB unwinding activity with ATP, dTTP, or AMPPNP. The author should add controls to verify that dsDNA lifetime were sufficiently long and not altered by nucleotide composition in the reaction buffer. In Fig. 5c, DnaB unwinds more slowly with dTTP than with ATP. How do the authors reconcile this with Fig. 4, which indicates preferential dTTP hydrolysis during DNA replication even when ATP is present?

We have added a control experiment to verify that the duplex substrate is stable under the nucleotide conditions used in the unwinding assay. In the absence of DnaB, the dsDNA substrate remains bound and does not dissociate over the duration of the experiment in buffers containing ATP, dTTP, and AMPPNP. These data are now included in Fig. 5c–e.

Indeed, unwinding (Fig. 5) and translocation assays[48] do not show the strong dTTP preference observed during replication, indicating that this is a replisome-engaged behaviour. We hypothesise that the cooperative behaviour with the other replisome proteins induces this change. This could include a conformational change in the DnaB hexamer, or the nucleotide-binding pocket.

6. In Lines 235-237: “Interestingly, the rate of unwinding (~1 nt/s) and the rate of strand displacement synthesis (~100 nt/s, Extended Data Fig. 8e) are both much smaller than the rate of DnaB-mediated replication (Fig. 1e, Extended Data Fig. 3b).” Please provide explicit methods and calculations for these rate estimates, including how ~1 nt/s and ~100 nt/s were derived, with references to the exact panels and fits in correct figures.

We have changed the numbers and added the requested references. The text now reads: “Interestingly, the rate of unwinding (~2 nt/s[11,59,60]) and the rate of strand-displacement synthesis (~100–150 nt/s, Extended Data Fig. 8e[61]) are both much smaller than the rate of DnaB-mediated replication (Fig. 1e, Extended Data Fig. 3b).”

7. In Lines 234-250, the author discussed the coupling between DnaB helicase and polymerase. Please clarify why this discussion appears at this point and how it relates to the central question of fuel choice (ATP vs. dTTP) during replication.

We have changed the text to better link the two concepts. See also our reply to Reviewer 2 comment 9.

8. Throughout the manuscript, DnaB was preloaded in ATP (required for loading per Extended Data Fig. 4), followed by ATP washout and initiation replication in dTTP-containing buffers. What is the physiological relevance of preferential dTTP use during elongation, while need ATP for loading? The author should expand the discussion to this point.

Helicase loading and replication elongation represent mechanistically distinct steps. In the presence of DnaC, ATP hydrolysis by DnaB has been shown to trigger DnaC dissociation and promote helicase ring closure (Rashid & Pageni *et al.*[48]). This observation raises the possibility that ATP may also facilitate a conformational transition between the DnaB loading state and an elongation-competent state, even in the absence of DnaC. We have added the following text to the discussion: "Notably, ATP is still required during the helicase loading step. While the mechanistic basis for this difference in nucleotide requirement remains unclear, ATP binding or hydrolysis may promote a conformational transition that places DnaB in an elongation-competent state."

9. The author could examine the structures of DnaB and other tested helicases regarding nucleotide binding-pocket permissiveness. Are there structural data supporting that the ATP-binding pocket can accommodate dTTP?

Yes, structural data from Rashid & Pangeni *et al.*[48] support that the nucleotide-binding pocket can accommodate dTTP. A 3.5 Å cryo-EM structure of hexameric DnaB bound to ssDNA and dTDP·AlFx shows that the thymine nucleotide adopts the same pose in the catalytic site as ATP.

10. Lines 171-174 and 217-218 cited reference 48 (an online conference abstract) to support their conclusions. Given that this is not a peer-reviewed publication and provides limited experimental and results details, the author might clarify this and temper the claims.

Reference 48 (Rashid & Pangeni *et al.*) is not a conference abstract. It is a full manuscript reporting similar observations that is concurrently undergoing peer review.

We thank all reviewers for their positive assessment of our work, and for the time and effort they invested in ensuring a rigorous peer-review process.

Reviewer #1 (Remarks to the Author):

The authors have sufficiently addressed most of my comments. I recommend accepting this manuscript for publication after the authors address these remaining minor comments:

1) The authors should explain the statistical tests carried out in the relevant figure captions and methods section as well, in addition to mentioning in the rebuttal letter.

We have now added the description of the statistical tests to the ‘Statistics and reproducibility’ section of the methods, as well as the relevant figure captions.

2) Fig. 4c caption: the authors should mention the ATP concentration (1.25 mM) to be consistent with Fig. 4b caption.

We have added the concentration to the caption for Fig. 4c.

3) I appreciate that the authors added discussions taking the in vivo concentrations of ATP and dTTP into account. The authors are correct that DnaB could use other nucleotides to drive its progression if dTTP levels drop. However, dTTP is obviously also essential for synthesizing nascent DNA, where dTTP depletion could lead to replication stress. I believe it is still one step too far to claim that dTTP hydrolysis is the main driving force of E. coli DNA replication in vivo, as suggested by the first sentence of Discussion. The in vivo scenario might be more complex and dynamic than that, considering the flex-fuel behavior of DnaB. I suggest that the authors temper down this claim.

We have added the following text to the first paragraph of the discussion: “However, the exact contribution of ATP and dTTP hydrolysis to replisome progression in vivo remains to be established.”

Reviewer #2 (Remarks to the Author):

I co-reviewed this manuscript with one of the reviewers who provided the listed reports. This is part of the Nature Communications initiative to facilitate training in peer review and to provide appropriate recognition for Early Career Researchers who co-review manuscripts.

Reviewer #3 (Remarks to the Author):

This is a much improved manuscript with better statistical comparisons and integrated discussions on nucleotide utilization within the E. coli replisome. The authors have included additional experiment to adequately respond to all the reviewer comments.

Reviewer #4 (Remarks to the Author):

The authors have improved the clarity of the manuscript and addressed all of my questions. It is acceptable for publication.
